# Uranium(iv) terminal hydrosulfido and sulfido complexes: insights into the nature of the uranium–sulfur bond†
†Electronic supplementary information (ESI) available: Full synthetic and experimental details, spectroscopic data for ^1^H NMR, SQUID, UV/vis/NIR, electrochemistry, and detailed X-ray crystallographic data. CCDC 1452972–1452974. For ESI and crystallographic data in CIF or other electronic format see DOI: 10.1039/c6sc00677a


**DOI:** 10.1039/c6sc00677a

**Published:** 2016-05-10

**Authors:** Michael W. Rosenzweig, Andreas Scheurer, Carlos A. Lamsfus, Frank W. Heinemann, Laurent Maron, Julie Andrez, Marinella Mazzanti, Karsten Meyer

**Affiliations:** a Department of Chemistry and Pharmacy , Inorganic Chemistry , Friedrich-Alexander University Erlangen-Nürnberg , Egerlandstraße 1 , 91058 Erlangen , Germany . Email: karsten.meyer@fau.de; b Institut des Sciences et Ingénierie Chimiques , Ecole Polytechnique Fédérale de Lausanne (EPFL) , 1015 Lausanne , Switzerland; c LPCNO , Université de Toulouse , INSA Toulouse , 135 Avenue de Rangueil , 31077 Toulouse , France

## Abstract

Herein, we report the synthesis and characterization of a series of terminal uranium(iv) hydrosulfido and sulfido complexes, supported by the hexadentate, tacn-based ligand framework (^Ad,Me^ArO)_3_tacn^3–^ (= trianion of 1,4,7-tris(3-(1-adamantyl)-5-methyl-2-hydroxybenzyl)-1,4,7-triazacyclononane). The hydrosulfido complex [((^Ad,Me^ArO)_3_tacn)U–SH] (**2**) is obtained from the reaction of H_2_S with the uranium(iii) starting material [((^Ad,Me^ArO)_3_tacn)U] (**1**) in THF. Subsequent deprotonation with potassium bis(trimethylsilyl)amide yields the mononuclear uranium(iv) sulfido species in good yields. With the aid of dibenzo-18-crown-6 and 2.2.2-cryptand, it was possible to isolate a terminal sulfido species, capped by the potassium counter ion, and a “free” terminal sulfido species with a well separated cation/anion pair. Spectroscopic and computational analyses provided insights into the nature of the uranium–sulfur bond in these complexes.

## Abstract 2

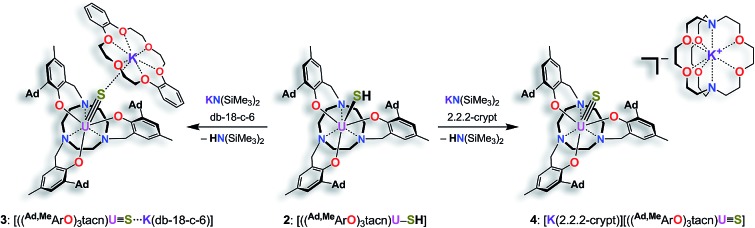
We report the synthesis and characterization of terminal uranium(iv) hydrosulfido and sulfido complexes, supported by the hexadentate, tacn-based ligand (^Ad,Me^ArO)_3_tacn^3–^.

## Introduction

The anhydrous coordination chemistry of the light actinides has become an active field of research since the discovery of suitable starting materials.^1^–^3^ Research has mainly focused on the chemistry of the mildly radioactive isotopes of uranium and thorium, rendering the resulting complexes suitable for practical applications ranging from catalysis to materials science.^4^–^13^ In contrast to the extensive uranium coordination chemistry with hard oxygen and nitrogen based ligands, bonding between uranium and the heavier chalcogens (S, Se, Te) was assumed to be disfavored due to the hard–soft mismatch. With the synthesis of stable uranium thiolate complexes in the late 1950s this theory was revoked^14^ and soft chalcogen containing ligands were developed for the selective complexation and separation of actinides and lanthanides in spent nuclear fuel. The selective complexation of 5f metal ions compared to 4f ions is based on the fundamentally different bonding and degree of covalency of the actinide *versus* lanthanide chalcogen bonds.^15^–^18^ In recent years, the number of actinide compounds with soft chalcogen ligands has been increasing steadily.^19^ DFT calculations of compounds with uranium–sulfur single bonds reveal this bond to be strongly polarized, thus essentially ionic in nature, whereas uranium–chalcogen multiple bonding is considered to be more covalent. Considerable academic as well as industrial interest in uranium chalcogenide multiple bonding has triggered efforts to synthesize well-defined mononuclear U=E compounds (E = O, S, *etc.*), enabling a more detailed insight into the electronic structure and degree of covalency in this structural motif.^20^–^32^

In contrast to the rapidly increasing number of reported terminal uranium oxo complexes,^20^–^26^ the number of fully characterized terminal uranium sulfido complexes remains scarce.^27^,^29^,^32^ This is likely due to the proclivity of uranium(iii) to undergo one electron oxidation resulting in dinuclear, sulfido-bridged diuranium(iv/iv) complexes rather than stabilizing the terminal sulfido ligand, S^2–^.^28^,^33^–^35^ Recently, our group established a facile synthetic route to mononuclear uranium(iv) hydrochalcogenido complexes employing H_2_E (E = S, Se, and Te) as the chalcogenido ligand source.^36^ Analogous to other known examples in transition metal chemistry, these uranium hydrosulfido complexes are suitable precursor molecules for the high-yield synthesis of terminal chalcogenido complexes, since the proton can be conveniently removed.^37^,^38^ Additionally, the U–EH species can be seen as “proton-capped” terminal chalcogenido complexes and spectroscopic comparison to the analogous, truly terminal species provides unique insight into the nature of the chemical bond between uranium and the soft chalcogenido ligand.^27^–^29^,^32^ Until today, there is only one structurally characterized uranium hydrosulfido complex reported in the literature, namely [((^Ad,Me^ArO)_3_N)U–SH(DME)] (with (^Ad,Me^ArO)_3_N^3–^ = trianion of tris(2-hydroxy-3-(1-adamantyl)-5-methylbenzyl)amine). Due to their potential application as catalysts, transition metal hydrochalcogenido complexes (E = O, S, Se, and Te) have received considerable interest in recent years.^37^–^45^ Most recently a uranium(iv) hydroxo complex, namely [((^Ad,Me^ArO)_3_mes)-U–OH], was found to be the key intermediate in the electrocatalytic production of dihydrogen from water.^9^

## Results and discussion

We previously demonstrated that the uranium(iii) complexes [((^*t*Bu,*t*Bu^ArO)_3_tacn)U] (with (^*t*Bu,*t*Bu^ArO)_3_tacn^3–^ = trianion of 1,4,7-tris-(3,5-di-*tert*-butyl-2-hydroxybenzyl)-1,4,7-triazacyclononane) and [((^Ad,Me^ArO)_3_N)U(DME)] (with ^Ad,Me^ArO)_3_N^3–^ = trianion of tris(2-hydroxy-3-(1-adamantyl)-5-methylbenzyl)amine) efficiently activate the elemental chalcogens (E = O, S, Se, and Te)^34^ as well as their hydrogen chalcogenides H_2_E.^36^ Uranium-mediated reductive transformations with the employed ligand systems, however, did not facilitate the formation of terminal uranium chalcogenido complexes. Instead, dimerization of the complexes *via* (poly-)chalcogenido as well as bis-hydrochalcogenido bridges was observed.^34^,^36^,^46^ In order to prevent dimerization reactions, we made use of a well-established tacn anchored ligand, the sterically encumbered adamantyl derivative (^Ad,R^ArO)_3_tacn)^3–^ (R = *tert-*butyl, methyl).^29^ Accordingly, the uranium(iii) precursor [((^Ad,Me^ArO)_3_tacn)U] (**1**) ((^Ad,Me^ArO)_3_tacn^3–^ = trianion of 1,4,7-tris(3-(1-adamantyl)-5-methyl-2-hydroxybenzyl)-1,4,7-triazacyclononane) allowed for synthesis of the here reported monomeric uranium (hydro-) chalcogenido complexes. More importantly, the bulky adamantyl groups effectively prevent dimerization upon deprotonation of the SH^–^ ligand; thus, yielding the targeted uranium terminal sulfido complex for direct comparison to the bonding situation in U complexes with η^1^-SH and η^1^-S ligands. The presence of crown ethers or cryptands in the deprotonation step not only increases the solubility of the formed metal salts, but additionally allows for the quantitative evaluation of the bonding situation in a U–S–H *versus* a U–S···K complex.

### Syntheses and molecular structures of terminal uranium(iv) hydrosulfido and sulfido complexes

**Scheme 1:**
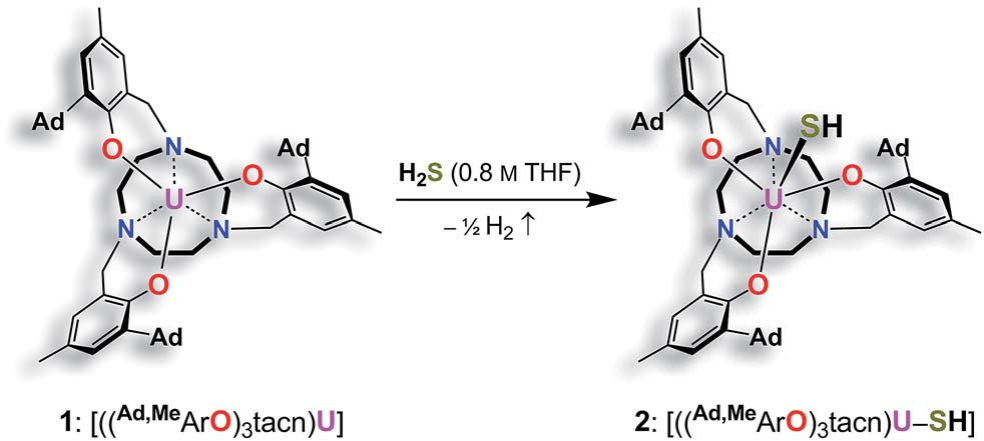
Synthesis of the uranium(iv) hydrosulfido complex [((^Ad,Me^ArO)_3_tacn)U–SH] (**2**).

Reaction of the uranium(iii) complex [((^Ad,Me^ArO)_3_tacn)U] (**1**) with various S atom transfer reagents, such as Ph_3_P=S or elemental sulfur, does not yield terminal U≡S complexes. Either a reaction was not observed at all or an intractable mixture of compounds without any isolable product was received. Finally, the synthesis of terminal uranium(iv) hydrosulfido and sulfido complexes was successfully achieved by treatment of complex **1** with one equivalent of H_2_S. The dropwise addition of 0.8 M H_2_S in THF to a red-brown solution of **1** in THF reproducibly affords the uranium(iv) hydrosulfido complex [((^Ad,Me^ArO)_3_tacn)U–SH] (**2**) in excellent yields with concomitant evolution of H_2_ gas (Scheme 1). After stirring for two hours, the blue-green precipitate was collected by filtration to afford the analytically pure complex **2** in 82% yield. The solid-state molecular structure of **2**·3.25 CH_2_Cl_2_ was unambiguously established by single-crystal X-ray diffraction analysis of the light green prisms, obtained by *n*-pentane diffusion into a concentrated DCM solution of **2**.

**Fig. 1:**
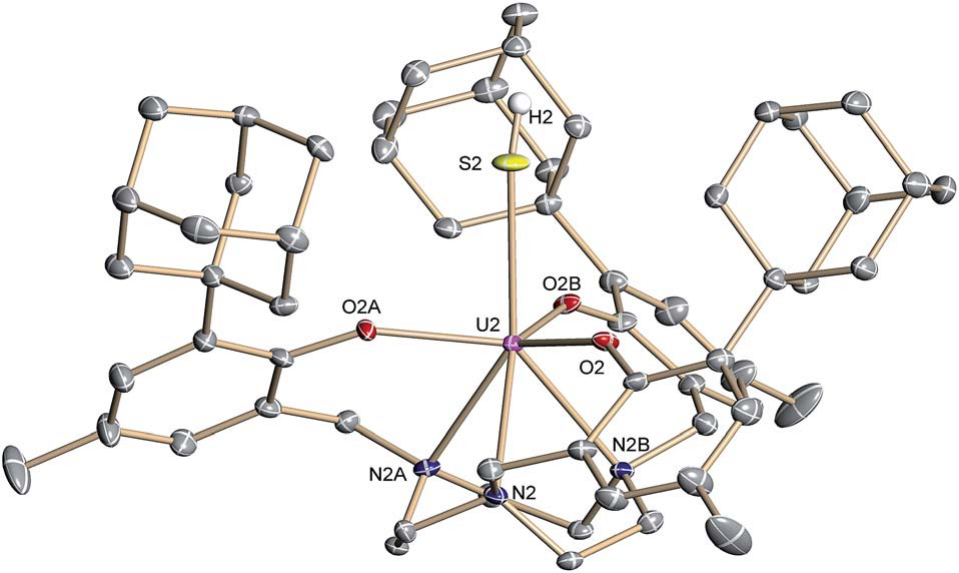
Molecular structure of the uranium(iv) hydrosulfido complex [((^Ad,Me^ArO)_3_tacn)U–SH] (**2**) with the chalcogen-bound H atom located in the difference Fourier map. All other hydrogen atoms and the solvent molecules are omitted for clarity (50% probability ellipsoids).

Complex **2**·3.25 CH_2_Cl_2_ crystallizes in the chiral hexagonal space group *P*6_3_ with two independent molecules per asymmetric unit (*Z* = 4). The mononuclear complex [((^Ad,Me^ArO)_3_tacn)U–SH] exhibits a seven-coordinate uranium ion in a face-capped octahedral coordination environment (Fig. 1).^47^ The U–S bond lengths of the two independent molecules in the crystals of **2** were determined to be 2.844(4) and 2.775(2) Å, respectively. This is in good agreement with other reported uranium–sulfur single bonds (2.588(1)–2.907(3) Å)^36^ but distinctly longer than published uranium species with terminal sulfido ligands (2.382(11)–2.481(1) Å).^27^–^29^,^32^ The SH^–^ ligand is situated on the *C*_3_ axis of the molecule in the axial position, *trans* to the tacn anchor. Since the chalcogen-bound H atom could be located in the difference Fourier map, the U–S–H angle was determined to be 152° and 156°, respectively. The U–O_aryloxide_ distances are 2.152(4) Å and 2.188(3) Å, respectively, and the U–N_tacn_ bond lengths are 2.680(5) Å and 2.650(4) Å. The uranium out-of-plane shift (U_oop_), defined by the displacement of the uranium ion below the plane of the three aryloxide oxygen atoms, was measured to be –0.282 and –0.268 Å, respectively. All these parameters are in good agreement with other uranium(iv) complexes supported by the (^R,R′^ArO)_3_tacn^3–^ ligand system (R = 1-adamantyl, *tert*-butyl, neo-pentyl; R′ = *tert*-butyl, methyl).^22^,^48^–^52^

**Scheme 2:**
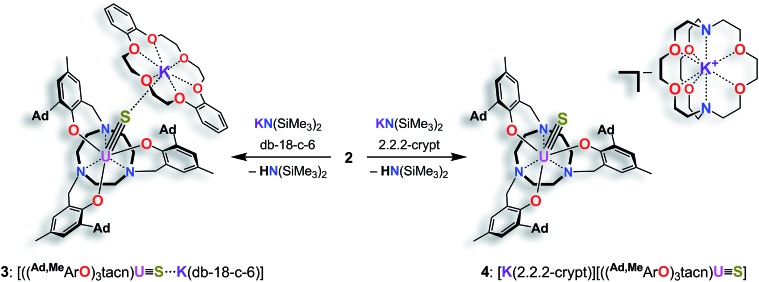
Synthesis of the terminal uranium(iv) sulfido complexes [((^Ad,Me^ArO)_3_tacn)U≡S···K(db-18-c-6)] (**3**) and [K(2.2.2-crypt)][((^Ad,Me^ArO)_3_tacn)U≡S] (**4**).

**Fig. 2:**
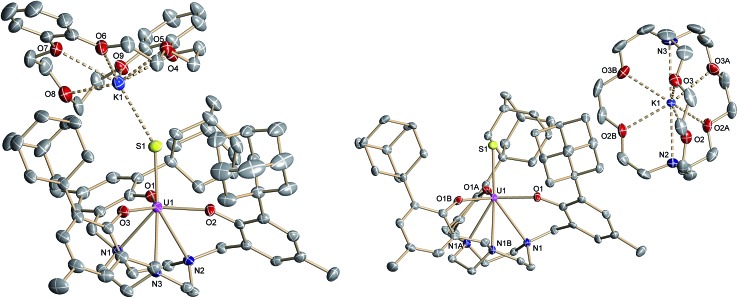
Molecular structure of the uranium(iv) sulfido complex [((^Ad,Me^ArO)_3_tacn)U≡S···K(db-18-c-6)] (**3**) (left), and [K(2.2.2-crypt)][((^Ad,Me^ArO)_3_tacn)U≡S] (**4**) (right). All hydrogen atoms and the solvent molecules are omitted for clarity (50% probability ellipsoids).

In order to obtain a terminal uranium(iv) sulfido species, complex **2** was treated with potassium bis(trimethylsilyl)amide in THF to deprotonate the –SH moiety. In order to encapsulate the potassium counterion, the reaction was performed in the presence of either dibenzo-18-crown-6 (= 6,7,9,10,17,18,20,21-octahydro-dibenzo[*b*,*k*]-[1,4,7,10,13,16]hexaoxacyclooctadecine; db-18-c-6) or 2.2.2-cryptand (= 4,7,13,16,21,24-hexaoxa-1,10-diazabicyclo[8.8.8]-hexacosane; 2.2.2-crypt) (Scheme 2). Single-crystal X-ray crystallographic structure determinations of the resulting orange products [((^Ad,Me^ArO)_3_tacn)U≡S···K(db-18-c-6)] (**3**) and [K(2.2.2-crypt)][((^Ad,Me^ArO)_3_tacn)U≡S] (**4**) were carried out. The uranium(iv) sulfido complex **3**·0.62 benzene·0.38 Et_2_O crystallizes in the monoclinic space group *P*2_1_/*c* with one molecule per asymmetric unit, whereas **4** crystallizes in the chiral, hexagonal space group *P*6_3_ with a third of one independent molecules per asymmetric unit. Both the uranium complex and the [K(2.2.2-crypt)] moiety were found on a crystallographic threefold axes. As anticipated, the sulfido ligand of the uranium(iv) complex [((^Ad,Me^ArO)_3_tacn)U≡S···K(db-18-c-6)] (**3**) is capped by the [K(db-18-c-6)]^+^ cation, whereas complex [K(2.2.2-crypt)][((^Ad,Me^ArO)_3_tacn)U≡S] (**4**) features a genuine terminal sulfido ligand with the [K(2.2.2-crypt)]^+^ cation in the outer coordination sphere of the complex anion (Fig. 2).

Like complex **2**, U^IV^ complex **3** features a seven-coordinate uranium ion with the sulfido ligand occupying the axial position. The S–K distance is 3.136(1) Å, demonstrating a bonding interaction between the S^2–^ ligand and the K^+^ counter ion (Fig. 2, left). The U–S bond length is 2.507(1) Å, which is significantly shorter compared to [((^Ad,Me^ArO)_3_tacn)U–SH] (**2**, d(U–SH)_av_ = 2.810(4) Å), but slightly longer than those of other reported uranium(iv) sulfido complexes (2.442(2)–2.4805(5) Å).^27^–^29^,^32^ While the ^1^H NMR spectrum of **3** reveals a *C*_3_-symmetrical molecule in solution (*vide infra*), coordination of the [K(db-18-c-6)]^+^ crown ether leads to a loss of *C*_3_ symmetry in the crystal structure. The average U–O_aryloxide_ distance of 2.219 Å and the mean U–N_tacn_ bond length of 2.819 Å are slightly longer compared to U^IV^ hydrosulfido complex **2**. Interestingly, the U out-of-plane shift (U_oop_) significantly decreases from –0.275 in **2** to –0.055 Å in **3**; hence, the uranium center is positioned almost perfectly in the plane of the three oxygen donors. This observation is quite unusual for uranium(iv) ions in the tacn-based ligand system, and is typically only seen for high-valent U^V^ and U^VI^ complexes with strong π-donor ligands, such as the oxo and isoelectronic imido functionality.^22^,^49^ However, as shown before, the U out-of-plane shift correlates well with the degree of U–L multiple bond character and bond strength; and thus, might be indicative of significant multiple bonding and covalent character of the U–S bond in **3** (*vide infra*).^22^,^53^

The connectivity of the N_3_O_3_S ligand donor set in the anionic complex [((^Ad,Me^ArO)_3_tacn)U≡S]^–^ (**4**)^–^ is analogous to that found for complex **3**. In the case of **4**, however, the potassium cation is encapsulated by the sterically encumbered 2.2.2-cryptand and located in the outer coordination sphere of the anionic U^IV^ complex, leading to a discrete ion pair with isolated [K(2.2.2-crypt)]^+^ cations and [((^Ad,Me^ArO)_3_tacn)U≡S]^–^ anions (Fig. 2, right). Surprisingly, although the sulfido ligand is no longer engaged in cationic interactions, the tetravalent complex **4** exhibits a slightly longer uranium–sulfido distance of 2.536(2) Å and—along with the longer U–S distance—a slightly but noticeably larger U_oop_ of –0.086 Å compared to **3** (d(U–S)_av_ = 2.507(1) and U_oop_ = –0.055 Å). It is suggested that the diphenyl-18-crown-6 moiety exerts a considerable steric strain that might push the sulfur atom slightly deeper into the cavity of the [((^Ad^ArO)_3_tacn)U] moiety, while at the same time, the uranium reduces its negative out-of-plane shift and moves closer to the sulfur atom in order to accommodate the sterically demanding potassium diphenyl-18-crown-6 moiety in the complex periphery. In addition, the seven-coordinate uranium center is chiral with an idealized *C*_3_ symmetry, affording a racemate of complex **4**. After crystallization, a conglomerate of enantiomerically pure crystals was found for **4** with an A-configuration of the uranium center in the analyzed crystal.^47^,^51^ Complexes **2–4** are stable in the solid form or in THF solution for at least 3 weeks without any notable decomposition.

### ^1^H NMR spectroscopy

^1^H NMR spectroscopy shows that compounds **2–4** possess *C*_3_ symmetry in solution, induced by coordination of the tacn ligand to the metal center with the hydrosulfido/sulfido ligand situated in the axial position on the *C*_3_ axis (see ESI†). Hence, complexes **2–4** are chiral in solution and the methylene protons of the tacn system as well as the benzyl groups are diastereotopic. Hydrosulfido complex **2** recorded in pyridine-*d*_5_ at 25 °C features 15 paramagnetically broadened and shifted signals from 10.31 to –32.84 ppm assigned to the 79 protons of the complex. The sulfur-bound hydrogen was assigned by integration to the broadened resonance at –32.84 ppm. The ^1^H NMR spectra of compounds **3** and **4** exhibit 14 proton signals in the range of 107 to –118 ppm, which could not be unequivocally assigned to certain protons of the complex. Furthermore, four additional resonances assigned to the [db-18-c-6] crown ether as well as three resonances for 2.2.2-crypt were observed. Apart from the crown ether/cryptand signals, the spectra of both terminal sulfido complexes **3** and **4** are almost perfectly superimposable, with only slight differences in the chemical shifts. It is noteworthy that—in contrast to the coronate signals of **3**—the cryptate ^1^H-NMR signals of **4** are not paramagnetically shifted, indicating a separated ion pair in solution. Interestingly, the solid state structure of **3** displays the sulfido ligand capped by the potassium counter ion with a U–S–K angle of 142°, thereby breaking the *C*_3_ symmetry of the molecule. However, ^1^H NMR spectroscopy clearly reveals a threefold symmetrical species in solution and the paramagnetically shifted coronate signals prove the sustained S···K interaction.^54^ Low-temperature VT-NMR measurements were performed on **3** (+20 °C to –80 °C in THF-*d*_8_) in order to investigate the complexes' symmetry at low temperatures. Upon cooling the ^1^H-NMR signals broaden and shift, but coalescence is not observed. The VT-NMR experiments thus suggest that, in solution, the crown ether complexated potassium ion remains in the vicinity of the paramagnetic uranium complex anion. It is further suggested that the potassium crown ether moiety fluctuates around the anion's threefold axis faster than the NMR timescale; even at very low temperatures. As a consequence, the U–S bond distance of **3** relaxes in solution, leading to a weaker uranium–sulfur bonding interaction (see computational analysis in the gas-phase, *vide infra*).

### Absorption spectroscopy

**Fig. 3:**
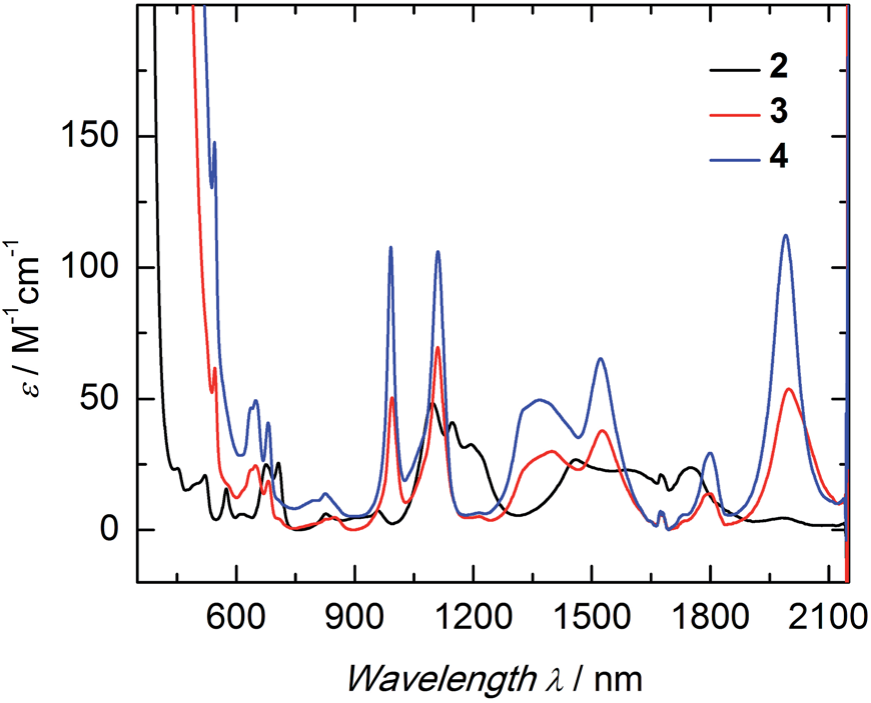
Electronic absorption spectra of the uranium hydrosulfido complex [((^Ad,Me^ArO)_3_tacn)U–SH] (**2**, black) and terminal sulfido complexes [((^Ad,Me^ArO)_3_tacn)U≡S···K(db-18-c-6)] (**3**, red) and [K(2.2.2-crypt)][((^Ad,Me^ArO)_3_tacn)U≡S] (**4**, blue); all complexes 5 mM in pyridine, measured at RT.

The electronic structure of complexes **2–4** was studied by UV/vis near-infrared spectroscopy. In the high-energy region (*λ* < 600 nm) broad and rather intense ligand-based absorption bands as well as charge-transfer transitions are observed. In the UV region of the spectra, all three complexes exhibit an absorption band at 298 nm with an extremely high extinction coefficient (Fig. S14†). This spin- and parity-allowed transition most likely arises from ligand π–π* transitions.^55^ Based on the assumption that the sulfido ligand has a certain degree of π-bonding character, this intensified electronic interaction between metal center and ancillary ligand should also be reflected in the electronic absorption spectrum. Indeed, both sulfido complexes **3** and **4** possess an additional absorption band in the visible region at 524 nm (*ε* = 190 M^–1^ cm^–1^ (**3**); 300 M^–1^ cm^–1^ (**4**)), absent in **2**. This absorption band is most likely due to a metal to ligand charge-transfer transition (MLCT) of a metal-centered 5f electron into a sulfido-based orbital.^56^ The unusually low intensity of this spin- and parity-allowed transition can be explained by the poor overlap of the diffuse 5f orbitals with the ligand orbital.^57^ This MLCT transition in the visible region is likely to be responsible for the color differences of the orange sulfido complexes **3** and **4** compared to the pale blue-green color of **2**. The visible near-IR electronic absorption spectra of complexes **2–4** in pyridine (5 mM) are shown in Fig. 3. Uranium(iv) complexes possess a 5f^2^ electron configuration, and therefore display rather complicated electronic absorption spectra with multiple low-intensity absorption bands and fine structure in the vis/NIR region.^58^ However, the Laporte forbidden f–f transitions with small extinction coefficients (*ε* = 6–116 M^–1^ cm^–1^) give rise to signature absorption bands characteristic of tetravalent uranium complexes. This is particularly true for a series of complexes, in which the symmetry around the metal center remains constant (*C*_3_ for all complexes in solution) and the core structure is dominated by the ((ArO)_3_tacn)^3–^ chelate, and thus, quite similar.^55^,^59^ The terminal sulfido complexes **3** and **4** exhibit 10 absorption bands with nearly identical absorption patterns in the vis/NIR region between 540 and 2100 nm, with three relatively strong absorption peaks at around 990, 1111, and 1990 nm. Noticeably, the molar extinction coefficients observed in the spectra of the separate ion pair **4** are consistently larger than those of complex **3**, with the capped terminal sulfido ligand. The NIR spectra have been reproduced multiple times and the differences in extinction coefficient are significantly larger than the experimental error. Further inspection of the NIR spectra reveals approximately equal line width for the absorption bands in complexes **3** and **4**; thus, excluding an intensity stealing mechanism.‡
‡Complex **3**: FWHM_994 nm_ = 26 nm, FWHM_1111 nm_ = 40 nm, FWHM_1998 nm_ = 106 nm; complex **4**: FWHM_991 nm_ = 24 nm, FWHM_1111 nm_ = 38 nm, FWHM_1990 nm_ = 76 nm. A reduced symmetry also cannot account for different extinction coefficients in **3** and **4**, since both possess *C*_3_ symmetry in solution (as established by (VT) ^1^H NMR spectroscopy, *vide supra*). However, it is worth noting that the timescale of electronic absorption spectroscopy is significantly shorter compared to proton NMR spectroscopy, therefore complex **3** could lose its *C*_3_ symmetry. Regardless, in the latter case, absorption bands of complex **3** should be more intense than those of complex **4**. Since the intensity of an electronic absorption band in the NIR region is considered indicative of the degree of covalency of the uranium ligand multiple bond in a conserved ligand field,^59^,^60^ the spectral data imply a less covalent bonding interaction for the potassium-capped uranium(iv) sulfido complex, **3**. This is in contrast to the shorter U≡S bond observed in the solid-state structure of **3** implying a stronger, more covalent bond compared to **4**. Therefore, one can only conclude—and reiterate—that the mere bond distance is not a valid measure of covalency.

Hydrosulfido complex **2** shows about the same number of absorption bands as **3** and **4**, but the f–f transitions occur at slightly different energies and a charge-transfer transition is not observed. Additionally, the intensities of the bands are significantly lower (*ε* = 6–48 M^–1^ cm^–1^) and, in accordance with the lack of charge-transfer transitions, indicate the presence of a ligand with predominantly σ-donor character.

### Magnetic investigations

**Fig. 4:**
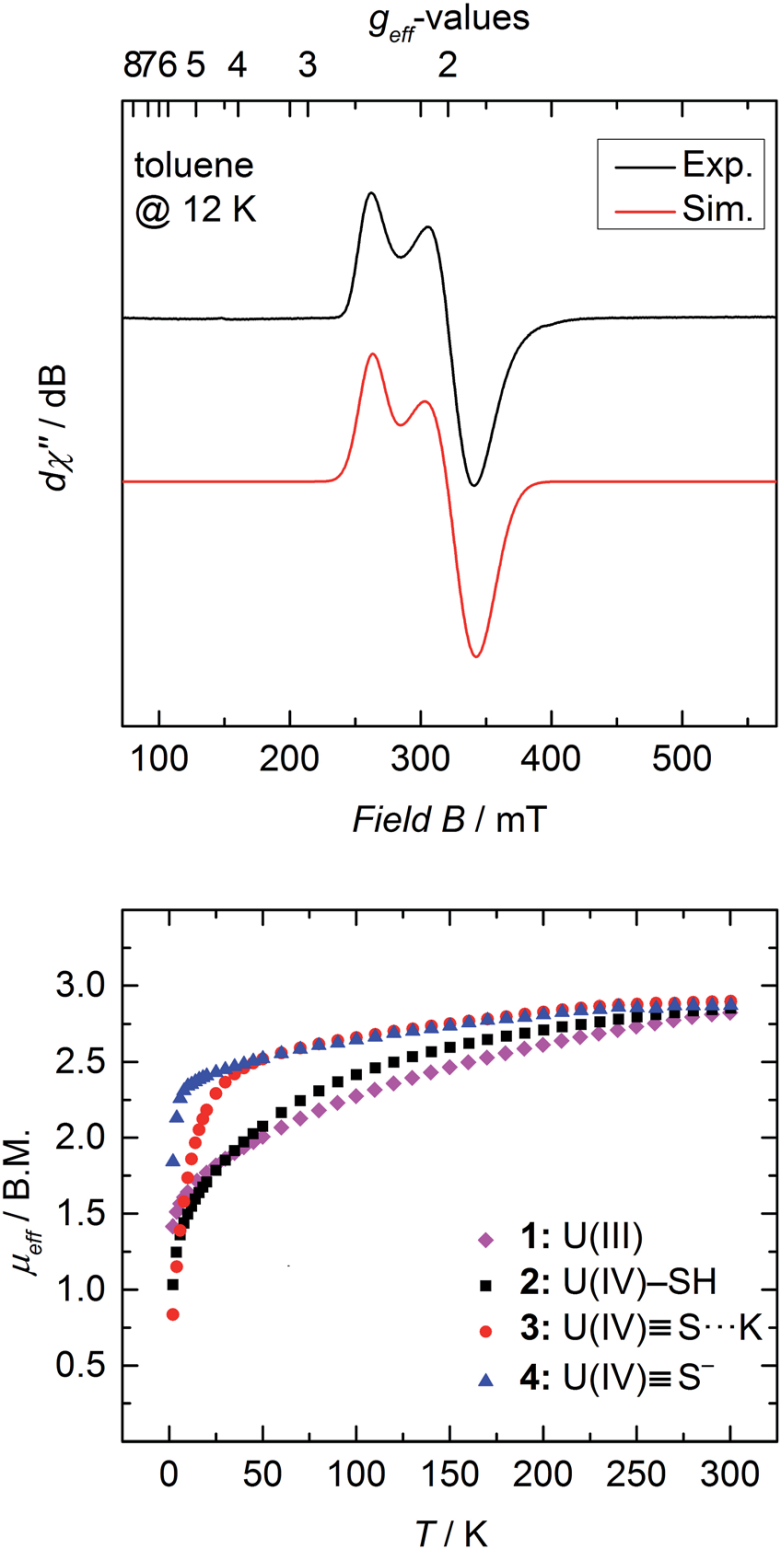
Top: X-band EPR spectrum of **1** recorded in toluene glass at 12 K. Experimental conditions: microwave frequency, 8.9641 GHz; power, 0.0997 mW; modulation, 1 mT. The spectrum was simulated with *g*_⊥_ = 1.912 and *g*_‖_ = 2.421 and Gaussian lines with *W*_⊥_ = 20.1 and *W*_‖_ = 11.4 mT; bottom: temperature dependent SQUID magnetometry of complexes [((^Ad,Me^ArO)_3_tacn)U] (**1**, magenta diamonds), [((^Ad,Me^ArO)_3_tacn)U–SH] (**2**, black squares), [((^Ad,Me^ArO)_3_tacn)U≡S···K(db-18-c-6)] (**3**, red circles), and [K(2.2.2-crypt)][((^Ad,Me^ArO)_3_tacn)U≡S] (**4**, blue triangles) plotted as *μ*_eff_*vs. T*.

SQUID magnetization measurements were carried out to study the temperature behavior of trivalent **1** and tetravalent **2–4** from 2 to 300 K (Fig. 4 bottom). Although the room temperature magnetic moments of transition metals and lanthanides can be accurately predicted by the spin-only (*μ*_S_) and total angular momentum approximations (*μ*_J_), respectively, there is currently no approximation to predict the magnetic moment for actinide coordination complexes, since ligand-field effects cannot be ignored and spin–orbit coupling is large.^50^,^61^,^62^ Tetravalent uranium ions possess an f^2^ valence electron configuration, which results in a non-magnetic ground state at very low temperatures; and consequently, strongly temperature-dependent magnetic moments, *μ*_eff_, with values typically ranging from 0.3 *μ*_B_ at 2 K to 2.8 *μ*_B_ at room temperature.^36^,^46^,^50^,^51^,^53^,^61^,^63^,^64^

In contrast, trivalent U^III^ ions (f^3^) possess a half integer spin with a doublet, EPR-active ground state (*g*_⊥_ = 1.912, *g*_‖_ = 2.421 (Fig. 4 top)) and should approach non-zero values at low temperatures.^53^,^65^ Accordingly, only the effective magnetic moment at low temperatures, as well as the temperature-dependency of the complexes, can provide reasonable hints to the ions' formal oxidation state. Complex **1** displays a strong temperature-dependent magnetic moment, varying from 1.42 *μ*_B_ at 2 K to 2.82 *μ*_B_ at room temperature. As already mentioned, the magnetic moment of 2.82 *μ*_B_ at room temperature is significantly smaller than the calculated moment (*μ*_J_ = 3.62 *μ*_B_), but the low temperature effective magnetic moment together with an EPR signal confirms a trivalent uranium ion in complex **1** (Fig. 4 top).

At room temperature, complexes **2–4** possess nearly the same magnetic moment with 2.85 *μ*_B_, 2.90 *μ*_B_, and 2.87 *μ*_B_, respectively, but show significantly different temperature-dependent behavior. These results support the notion that the room temperature magnetic moments cannot be used to determine the oxidation state of the uranium ion, since trivalent **1** at room temperature shows nearly the same (or even slightly lower) magnetic moment as tetravalent **2–4**. At 2 K, however, uranium(iv) complexes with the f^2^ ion typically show distinctively lower magnetic moments, which are due to the ions' non-magnetic singlet ground state.^61^ Complex **2** exhibits temperature-dependency overall typical for a uranium(iv) compound. The low magnetic moment, *μ*_eff_, of 1.03 *μ*_B_ at 2 K continually increases with increasing temperature. On the contrary, sulfido complexes **3** and **4** reveal an unusually strong temperature-dependency in the range of 2 to 50 K, with a subsequent moderate increase from 50 to 300 K. Notably, complex **3** shows a typically low magnetic moment of 0.84 *μ*_B_ at 2 K, whereas complex **4** possesses an unusually high *μ*_eff_ value of 1.84 *μ*_B_. Despite this high magnetic moment, complex **4** is EPR silent (in X band, between 300 and 5 K). Similar high magnetic moments have been observed for U^IV^ complexes with separate ion pairs like [Cp*_2_Co]-[U(O)(N(SiMe_3_)_2_)_3_],^27^ [Li(DME)]-[U(NC_5_H_10_)_5_],^66^ [Li(THF)_4_]-[U(CH_2_^*t*^Bu)_5_], and [Li(DME)_3_]-[U(CH_2_SiMe_3_)_5_].^67^

On the other hand, complex **2** possesses a more isolated magnetic ground state, where the higher magnetic states slowly become thermally accessible with increasing temperature. Hence, the low-lying magnetic states of complexes **3** and **4** appear to be closer in energy, and are already thermally accessible at temperatures below 50 K. Consequently, the magnetic moment increases rapidly from 2 to 50 K, and merely increases with increasing temperatures above 50 K. The intriguing difference in the temperature dependency of the magnetic moments of complexes **2–4** is due to the different crystal-field-splitting caused by the purely σ-type SH^–^*versus* the σ- and π-type S^2–^ ligands.^67^

### Electrochemistry

**Fig. 5:**
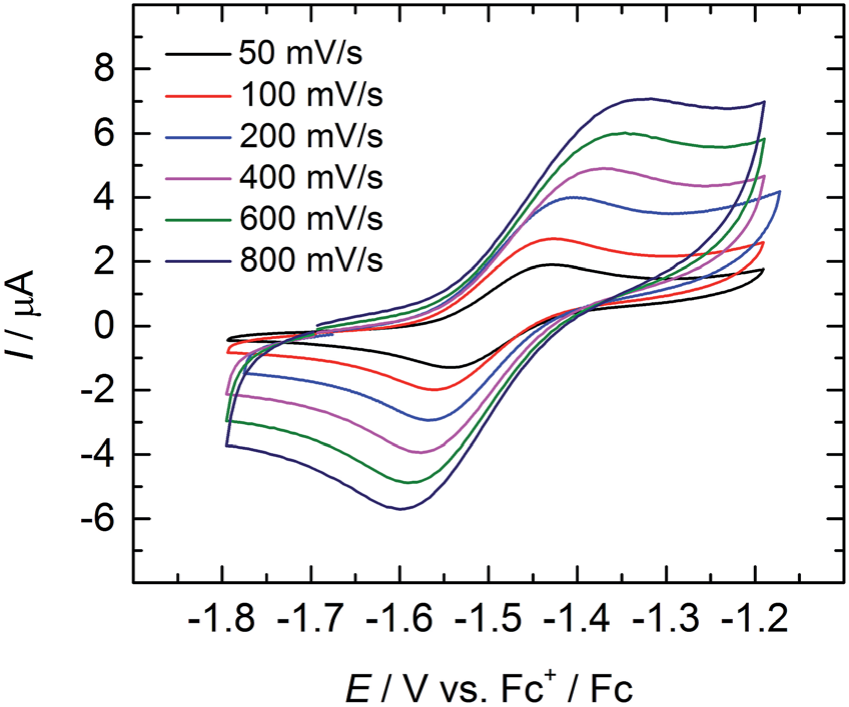
Quasi-reversible oxidation wave of **3** at different scan rates. Measurement conducted in THF with ∼0.1 M [N(*n*-Bu)_4_][BPh_4_] electrolyte, using Fc^+^/Fc couple as internal standard.

Cyclic and linear sweep voltammetry were performed on **3** in THF in the presence of ∼0.1 M [N(*n-*Bu)_4_][BPh_4_] electrolyte and the ferrocenium/ferrocene redox couple (Fc^+^/Fc) acting as internal standard. The cyclic voltammogram of **3** reveals a quasi-reversible redox process at a half-step potential, *E*_1/2_, of –1.494 V (Fig. 5). A positive current in the linear-sweep measurement confirms an oxidative process of the compound (see ESI†). Accordingly, this redox process is tentatively assigned to the uranium(iv/v) redox couple, with the half-step potential in the range of other published U^IV/V^ couples (–1.81 to 0.12 V *vs.* Fc^+^/Fc).^35^,^50^,^55^,^58^,^59^,^68^ Electrochemical data of uranium–chalcogenido complexes are exceedingly rare and reports on uranium–sulfido complexes are, to the best of our knowledge, not reported at all. However, dimeric uranium–oxo complex [{((^nP,Me^ArO)_3_tacn)U}_2_(μ-O)_2_] (nP = neopentyl) shows a comparable U^IV/V^ couple at a half-step potential of –1.55 V (*vs.* Fc^+^/Fc).^50^

Due to the poor solubility of **2** and **4** in polar solvents, such as THF, cyclic voltammetry experiments could not be performed with these complexes. Given the lack of characterized terminal uranium(v) sulfido complexes in the literature, and the expectation that the covalency of the uranium–chalcogenide bond increases with increasing valence,^62^ the chemical oxidation of **3** is desirable. Unfortunately, all attempts to chemically oxidize **3** and **4** have not yet been met with success and resulted in decomposition of the compounds.

### Theoretical studies

In order to gain further insight into the nature of the U–S bond, theoretical investigations were carried out on complexes **2–4**. Geometry optimizations were conducted on [((^Ad,Me^ArO)_3_tacn)U–SH] (**2**), [((^Ad,Me^ArO)_3_tacn)U≡S···K(db-18-c-6)] (**3**), and [K(2.2.2-crypt)][((^Ad,Me^ArO)_3_tacn)U≡S] (**4**) at the DFT level without any symmetry constraints. Subsequently, molecular orbital (MO) and natural bond orbital (NBO) analyses were performed.

**Fig. 6:**
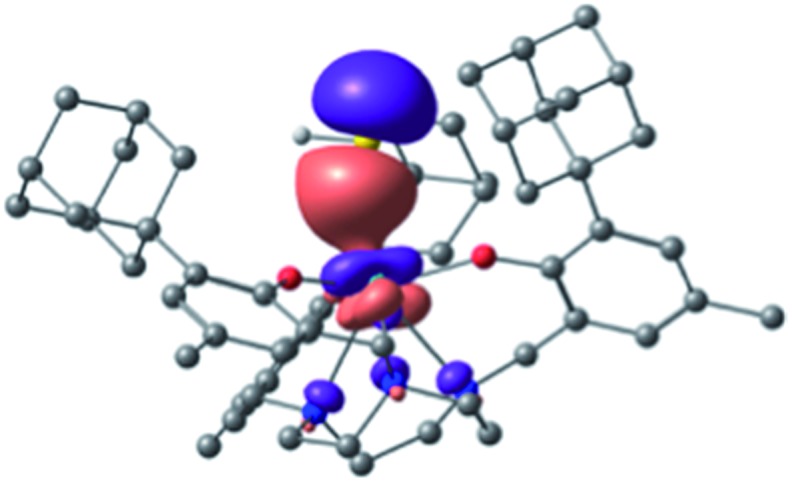
Bonding σ orbital of [((^Ad,Me^ArO)_3_tacn)U–SH] (**2**).

Initially, bond analysis was carried out on the hydrosulfido species **2**. The NBO analysis of **2** clearly indicates a single bond between U and S and a single bond between S and H (Wiberg bond indices (WBI) of 0.77 and 0.92, respectively). Accordingly, the molecular orbitals are consistent with a single U–S bond (Fig. 6), revealing the two non-bonding lone pairs to reside at the sulfur atom. A comparable terminal uranium(iv) hydrochalcogenido complex, namely [((*t*BuO)_3_SiO)_4_U(SH)]^–^ obtained by Andrez *et al.*, exhibits significant double bond character of the uranium sulfur interaction (determined by MO and WBI).^69^ In order to understand the origin of these electronic differences of **2** and [((*t*BuO)_3_SiO)_4_U(SH)]^–^, these two complexes were analyzed in more detail.

The U–S σ-bond of complex **2** is strongly polarized with 10% uranium and 90% sulfur orbital character. The metal orbital is a hybrid sdf orbital with 12% 7s, 38% 6d, and 50% 5f contribution. This is comparable to the hybrid orbital composition of the hydrosulfido complex [((*t*BuO)_3_SiO)_4_U(SH)]^–^ exhibiting a σ (and π) orbital with 14% uranium character (12% for the π) and a strongly hybridized orbital (10% 7s, 40% 6d and 50% 5f). As evidenced by the X-ray structure, the geometry of **2** differs significantly from the trigonal bipyramidal complex [((*t*BuO)_3_SiO)_4_U(SH)]^–^. The computational analysis suggests that the pyramidalized uranium ion of **2** has an efficient overlap with the N donor atoms of the tacn ring. This, in turn, results in a *trans*-effect reducing the U–SH bond strength, which is rather unusual for uranium complexes. In order to emphasize the importance of the *trans-*influence of the tacn ligand, a hypothetical tris(aryloxide) complex, 2* (without the triazacyclononane ligand) was also computed. Interestingly, this model complex adopts a tetrahedral geometry at the uranium center, and a U–S double bond character is found (see ESI† for the complete MO pictures and geometry).

**Fig. 7:**
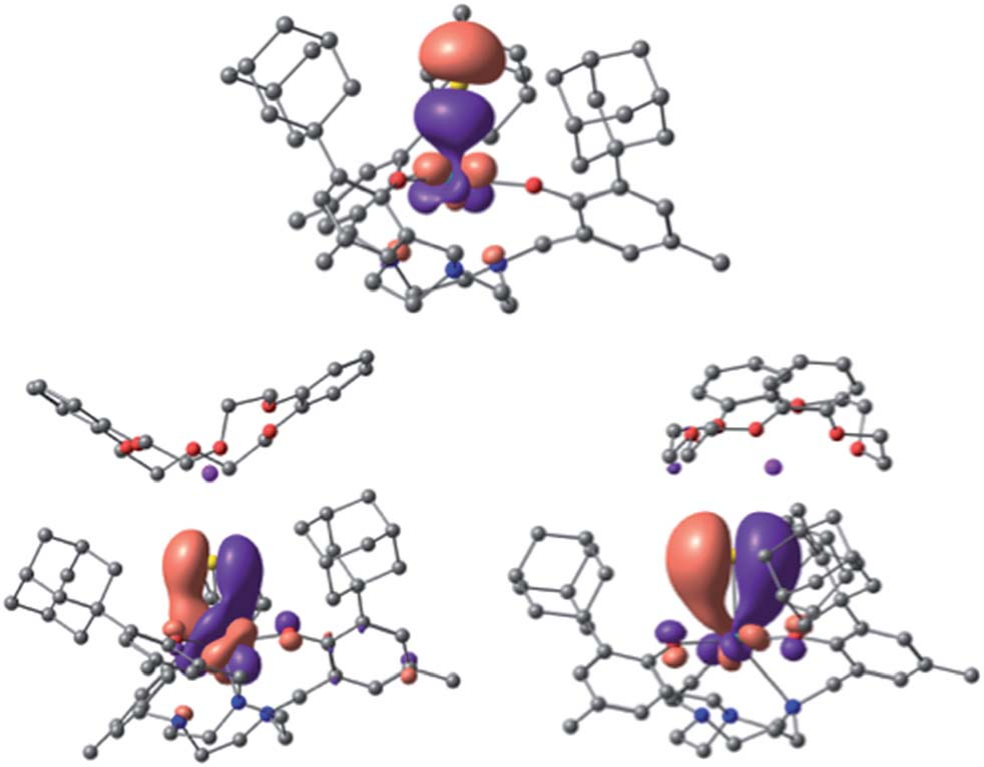
U–S bonding orbitals in [((^Ad,Me^ArO)_3_tacn)U≡S···K(db-18-c-6)] (**3**), with the σ (top) and the set of two π orbitals (bottom).

Bonding analysis of uranium(iv) complex **3** clearly reveals a formal U≡S triple bond with one σ and two π-type interactions (Fig. 7). The molecular structure of **3** (and **4**) illustrates that the uranium ion is situated almost perfectly in the trigonal plane of the three aryloxides with a weaker U–N_tacn_ interaction and more efficient uranium–sulfur orbital interaction resulting in the observed U–S multiple bond. NBO analysis shows that the U–S bond is strongly polarized with more than 75% charge on the sulfur. A σ-bond is formed by an sp orbital of sulfur (77%) and a d_z2_/f_z3_ hybrid orbital (41% 6d, 59% 5f) of uranium (23%), and two π orbitals are formed by the interaction of a p lone pair of sulfur (either p_*x*_ or p_*y*_, 77%) and a hybrid d_π_/f_π_ orbital (40% 6d, 60% 5f) of uranium (23%). This formal uranium sulfur triple bond is virtually unaffected by the minor interaction of the sulfido ligand with the potassium counterion (WBI of 0.1). To further substantiate the effect of the weakly associated K^+^ ion in **3**, the bonding analysis of **4** with an encrypted and well-isolated potassium ion was carried out. As expected, a triple bond between uranium and the sulfido ligand was found with the orbitals closely resembling those of **3** (see Fig. S17† for the MOs of **4**). The experimentally determined U–S bond length of **4** (without the S···K interaction) is elongated compared to **3**. However, this result is not reproduced by the calculations that show the bond in **3** to be slightly longer (0.02 Å) than in **4** (see Table S4 in ESI,† molecules calculated in the gas-phase).

In the calculation, a weak S···K interaction (10 kcal mol^–1^ at the second order donor–acceptor NBO) in **3** leads to a stronger negative charge on the sulfido ligand (–0.1 unit difference), which is formally interacting with two positively charged ions. Since the charge at the uranium ion is the same for **3** and **4**, the coordination of the potassium ion leads to a higher negative charge on the sulfido ligand in **3**, counterbalancing the charge. Consequently, a higher charge on the sulfido ligand in complex **3** leads to a smaller orbital overlap, and therefore less covalent interaction. In order to determine the nature of this discrepancy between experiment and theory, calculations were carried out on the putative anionic complex [((^Ad,Me^ArO)_3_tacn)U(S)]^–^ (**4**^–^). The bonding analysis confirmed the negligible influence of the K^+^ ion on the electronic structure of the U≡S bond of **3** and **4**, but not on the U–S bond length (see Fig. S18† for the MOs of the putative complex anion **4**^–^). Complex **4**^–^ possesses the shortest U–S distance, in line with the influence of the K^+^ bonding, but contrary to the bond lengths observed in the solid state (see Table S4 in the ESI†). Complex **3** was optimized taking dispersion interactions into account by applying the empirical Grimme corrections.^70^ This leads to a decrease in the U–S bond length by 0.02 Å in complex **3**. Hence, the computational analysis suggests that the experimentally observed shorter bond length of **3** is likely due to crystal packing effects that were not considered in the calculations (*vide supra*). Perrin *et al.* reported a similar effect for the distorted geometry of an amido lanthanide complex.^71^

Based on all of these results, we assign a significant degree of covalency to the U–S bond of complexes **2–4**. The uranium covalency contribution is defined by up to 60% 5f orbital character with the remainder being due to 7s and 6d orbital involvement. The dominating role of the latter orbitals is demonstrated by f-in-core calculations with the f-electrons included in the core shell configuration and unavailable for bonding. The results are essentially the same for complexes **2–4**. For instance, for **3**, a triple U≡S bond is obtained, which is strongly polarized towards S (between 70 and 75%) with hybrid s/d orbital involvement of the metal (roughly 80% 6d). Interestingly, the nature of the U≡S bond of complexes **3** and **4** appears to be quite similar to other computed uranium(iv) chalcogenido complexes with different supporting ligand systems.^72^,^73^ These results indicate that the ligand field, induced by the supporting ligand system, does not significantly affect the bonding within the U≡S entity, regardless of whether aryl-oxide, siloxide, or supporting amide ligands are applied. In all reported complexes, the geometries at the uranium center are either distorted tetrahedral or trigonal pyramidal. Quite surprisingly, the atomic 5f and 6d orbitals experience a very similar ligand field effect in all complexes.

In order to investigate the possible influence of the chalcogenido ligand, the bonding analyses of the oxo-homologs of **3** and **4**^–^ were carried out. Based on the report by Andersen on a Cp*_2_UO compound, a more ionic bonding description can be expected for the oxo complex.^74^ The NBO analysis is in line with a single U–O σ-bond (found for the second order donor–acceptor interaction of an sp-lone pair on O and an s/d/f hybrid orbital). The second order donor–acceptor calculation also hints at a small interaction between a π lone pair of O and an empty d/f orbital on U, but is too small in energy to be considered a bonding interaction (40 kcal mol^–1^, in line with a strong agostic interaction, see Table S4 ESI†). Hence, the oxo complexes are strongly ionic, whereas the sulfur analogs are more covalent. These results are in accordance with an increase in valence orbital energy of the heavier chalcogen homologs.

## Conclusion

In summary, we here present a new and high-yield synthetic protocol and the characterization of the terminal uranium hydrosulfido and sulfido complexes **2–4**, supported by the (^Ad,Me^ArO)_3_tacn^3–^ ligand system. Proton NMR spectroscopy reveals *C*_3_ symmetry of the complexes in solution, and the vis/NIR electronic absorption spectroscopy, together with the SQUID magnetization measurements, allow for the unambiguous assignment of the uranium ion to the +IV oxidation state. The differences in temperature-dependency of complexes **3** and **4** at low temperatures (*T* < 50 K) also suggest a significant influence of the potassium counter ion on the crystal field splitting of the terminal sulfido complexes as well as the nature of the U–S bond. DFT computational analyses further provided detailed insight into the bonding properties of complexes **2–4**, and reveal a non-negligible degree of covalency in the uranium–sulfur bond of **3** and **4**. This is supported by the complexes’ structural parameters, vis/NIR electronic absorption spectroscopy, and SQUID magnetometry. The electrochemical studies show that complex **3** can be electrochemically oxidized, most likely to a U^V^≡S species, which is expected to exhibit an even greater degree of covalency of the uranium sulfur bond.^62^ However, initial attempts to chemically oxidize and isolate a U(v) sulfido complex led to decomposition products.

The synthesis of a complete series of uranium(iv) complexes with terminal hydrochalcogenido and chalcogenido ligands is part of our on-going studies.

## Supplementary Material

Supplementary information

Crystal structure data
