# Supplementary material for: Uranium(iv) terminal hydrosulfido and sulfido complexes: insights into the nature of the uranium–sulfur bond
Source: Chem Sci. 2016 May 10;7(9):5857–66. doi: 10.1039/c6sc00677a (PMC6024247; doi:10.1039/c6sc00677a)
Supplement: Supplementary file 1 [file SC-007-C6SC00677A-s001.pdf]

Electronic Supplementary Material (ESI) for Chemical Science.
This journal is © The Royal Society of Chemistry 2016

Supporting Information for

Uranium(IV) Terminal Hydrosulfido and Sulfido Complexes: Insights into the Nature of the Uranium–Sulfur Bond

Michael W. Rosenzweig,[a] Andreas Scheurer,[a] Carlos A. Lamsfus,[c] Frank W. Heinemann,[a] Laurent Maron,[c] Julie Andrez,[b] Marinella Mazzanti[b] and Karsten Meyer*,[a]

[a.] *Department of Chemistry and Pharmacy, Inorganic Chemistry, Friedrich-Alexander University Erlangen-Nürnberg, Egerlandstraße 1, 91058 Erlangen, Germany.*
[b.] *Institut des Sciences et Ingénierie Chimiques, Ecole Polytechnique Fédérale de Lausanne (EPFL), 1015 Lausanne, Switzerland.*
[c.] *LPCNO, Université de Toulouse, INSA Toulouse, 135 Avenue de Rangueil, 31077 Toulouse, France.*

Table of Contents

Experimental Section....2
Ligand Synthesis....3
Complex Synthesis....3
Crystallographic Details....5
X-ray Crystal Structure Determination Details....5
Graphical representations....10
^{1}H NMR spectroscopy....13
IR spectroscopy....17
Electronic Absorption Spectroscopy....19
Magnetism....22
Computational Study....27
References....51

Experimental Section

General Methods. All air and moisture-sensitive experiments were performed under dry nitrogen atmosphere using standard Schlenk techniques or in MBraun inert-gas gloveboxes containing an atmosphere of purified dinitrogen. The glovebox is equipped with a –35 °C freezer. Solvents were purified using a two-column solid-state purification system (Glasscontour System Irvine, CA) and transferred to the glovebox without exposure to air, and stored over molecular sieves and sodium (where appropriate). All glassware was dried by storage in an oven overnight (min. 12 h) at a temperature of 125 °C.

^{1}H NMR spectra were recorded at room temperature on a JEOL ECX 400 (^{1}H: 399.78 MHz). Deuterated solvents were purchased from Acros Organics®. The chemical shift δ is quoted in ppm. Thereby, the ^{1}H signal of the remaining undeuterated part of the solvent acts as internal standard for ^{1}H spectra (δ = 7.16 (benzene-d_6), δ = 7.26 (chlorofrom-d_1), δ = 8.74 (pyridine-d_5))

Magnetism data of crystalline powdered samples (20 – 30 mg) were recorded with a SQUID magnetometer (Quantum Design) at 10 kOe at a temperature range of 2 – 300 K. Values of the magnetic susceptibility were corrected for the underlying diamagnetic increment (X_{dia} = –676.57 x 10^6 cm^3mol^{-1} (**1**), –713.50 x 10^6 cm^3mol^{-1} (**2**), –922.67 x 10^{-6} cm^3mol^{-1} (**3**), –951.29 x 10^{-6} cm^3mol^{-1} (**4**)) by using Pascal constants.[1] Samples used for magnetization measurements were checked for chemical composition and purity by ^{1}H NMR spectroscopy. Data reproducibility was also carried out on independently synthesized samples.

Elemental analyses (C, H, N, and S) were obtained from the Analytical Laboratories at the Friedrich-Alexander University Erlangen-Nürnberg (Erlangen, Germany) using Euro EA 3000 (Euro Vector).

IR spectroscopy was performed on Shimadzu Affinity-1 CE FTIR instrument from 350 to 4000 cm^{-1}. Solid samples were measured as potassium bromide pellets, which were produced inside an inert-gas glovebox. All spectra have been smoothed and are reported in $\tilde{\nu}$ (cm^{-1}). Intensities of peaks are reported as vs (very strong), s (strong), m (medium), and w (weak).

Electronic absorption spectroscopy was performed on Shimadzu UV-3600 in the indicated solvent from 200 to 2300 nm in air-tight quartz glass cuvettes

The electrochemistry was investigated, using a three-electrode setup with rotating glassy carbon working electrode and platinum rods as counter– and reference electrode. The potentiostat was an Autolab Type–III. The electrochemical cell was placed inside an inert–gas glovebox under nitrogen atmosphere and the samples were measured in 0.1 M electrolyte solutions of [N(*n*–Bu)$_4$][BPh$_4$] (purchased from Acros and used without further purification) in THF. The reported half-wave potential is referenced to the Fc^+/Fc redox couple, which was measured by adding ferrocene to the sample solution.

Starting Materials. 2-adamantyl-4-methylphenol was prepared according to literature procedure.[2] Precursor complex [U(N(SiMe$_3$)$_2$)$_3$] was prepared as described by Kiplinger and coworkers.[3] Potassium bis(trimethylsilyl)amide (95%), dibenzo-18-crown-6 (98%), 4,7,13,16,21,24-hexaoxa-1,10-diazabicyclo[8.8.8]hexacosane (98%) (2.2.2-crypt) , paraformaldehyde (reagent grade), and Hydrogen

sulfide (0.8 M in THF) were purchased from Sigma-Aldrich and used without further purification. H_2S gas is extremely toxic and should only be handled in a well-ventilated fumehood or glovebox.

Ligand Synthesis

A solution of tacn (2.59 g, n = 0.02 mol) and paraformaldehyde (3.00 g, 0.10 mol) in methanol (25 mL) was refluxed for 2 h. 2-adamantyl-4-methylphenol (17.0 g, n = 0.07 mol) and a few drops of conc. HCl were added until the solution turns yellow. The mixture was refluxed overnight (12 h) and allowed to cool to room temperature. The product was isolated by filtration. For purification, the white solid was stirred in methanol and put in a sonication bath for 10 h. The white solid was collected by filtration and washed with warm methanol and acetonitrile.

Yield: 9.20 g (n = 0.01 mol, 50 %).

^{1}H NMR (400 MHz, chloroform-*d*$_1$): δ [ppm] = 10.07 (br. s, 3H, OH), 6.99 (s, 3H, aryl-H), 6.57 (s, 3H, aryl-H), 3.68 (s, 6H, NCH_2Ph), 2.84 (s, 12H, NCH_2CH_2N), 2.24 (s, 9H,$PhCH_3$), 2.19 (s, 18H, Ad), 2.09 (s, 9H, Ad), 1.81 (s, 18H, Ad).

Elemental analysis (%) calcd for $(^{Ad,Me}ArOH)_3$tacn: C 80.67, H 9.15, N 4.71; found C 80.31, H 9.01, N 4.59.

Complex Synthesis

Synthesis of [((^{Ad,Me}ArO)$_3$tacn)U] (1). A purple solution of $[U(N(SiMe_3)_2)_3]$ (1.00 g, 1.4 mmol) in *n*-pentane (4 mL) was added to a colorless solution of 1,4,7-tris(3-adamantyl-5-methyl-2-hydroxybenzyl)-1,4,7-triazacyclononane) (1.25 g, 1.4 mmol) in benzene (3 mL). The solution immediately turns red-brown and was allowed to stir for 2 h to ensure complete reaction. The reaction mixture was concentrated and cooled in a freezer (-34 °C). The red-brown solid was collected by filtration, washed three times with a mixture of benzene/*n*-pentane (1:2), and dried *in vacuo*.

Yield: 1.34 g (n = 1.19 mmol, 85 %).

^{1}H NMR (400 MHz, benzene-*d*$_6$): δ [ppm] = 12.91 (s, 3H), 9.85 (s, 3H), 7.40 (s, 3H), 4.22 (s, 9H), 3.13 (s, 9H), 0.54 (s, 9H), 0.31 (s, 9H), –0.12 (d, *J* = 11.03 Hz, 9H), –1.65 (d, *J* = 11.03 Hz, 9H), –3.27 (d, *J* = 11.03 Hz, 3H), –5.30 (s, 3H), –5.95 (s, 3H), –9.03 (s, 3H), –21.73 (s, 3H).

Elemental analysis (%) calcd for [((^{Ad,Me}ArO)$_3$tacn)U]: C 63.93, H 6.97, N 3.73; found C 63.71, H 6.91, N 3.34.

Synthesis of [((^{Ad,Me}ArO)$_3$tacn)U–SH] (2). A vial was charged with [((^{Ad,Me}ArO)$_3$tacn)U] (166 mg, n = 0.15 mmol) in 3 mL THF and a solution of H_2S in THF (0.8 M, n = 0.15 mmol, 187 µL) was added dropwise. After 2 h a cyan precipitate formed and was collected by filtration. The solid was washed with cold THF a couple of times and dried under vacuum. Crystals suitable for X-ray diffraction were obtained by layering a concentrated solution of [((^{Ad,Me}ArO)$_3$tacn)U–SH] in DCM with *n*-pentane.

Yield: 140 mg (n = 0.12 mmol, 82%).

^{1}**H NMR** (400 MHz, pyridine-d_5): δ [ppm] = 10.31 (d, J = 14.7 Hz, 3H), 9.62 (s, 3H), 8.00 (s, 3H), 5.93 (s, 3H), 3.36 (s, 3H), 2.66 (d, J = 11.03 Hz, 9H), 2.38 (s, 9H), 1.62 – 1.57 (s, 18H), 1.41 (d, J = 11.03 Hz, 9H), 0.10 (d, J = 11.04 Hz, 9H), –2.95 (s, 3H), –4.83 (s, 3H), –18.13 (s, 3H), –32.84 (s, 1H, S**H**).

IR (KBr pellet): $\tilde{\nu}$ (cm^{-1}) = 2901 (vs), 2847 (vs), 1601 (w), 1462 (vs), 1443 (s), 1417 (m), 1373 (w), 1342 (m), 1304 (s), 1285 (m), 1250 (vs), 1223 (s), 1186 (w), 1161 (m), 1103 (s), 1065 (m), 1034 (m), 1007 (m), 972 (m), 887 (w), 860 (m), 825 (s), 806 (m), 779 (m), 756 (m), 702 (w), 679 (w), 602 (w), 575 (w), 525 (s), 451 (w), 416 (m).

Elemental analysis (%) calcd for [((^{Ad,Me}ArO)$_3$tacn)U–SH] · 2 THF: C 62.60, H 7.34, N 3.22, S 2.46; found C 62.70, H 7.28, N 3.24, S 1.91.

Synthesis of [((^{Ad,Me}ArO)$_3$tacn)U≡S···K(db-18-c-6)] (3). A vial was charged with [((^{Ad,Me}ArO)$_3$tacn)U–SH] (50 mg, n = 0.04 mmol) and 2 mL of pyridine, resulting a cloudy cyan solution. First, dibenzo-18-crown-6 (14 mg, n = 0.04 mmol) was dissolved in 1 mL pyridine and added to the reaction solution, followed by the dropwise addition of K(N(SiMe$_3$)$_2$) (8 mg, n = 0.04 mmol) in 1 mL pyridine. The solution turns immediately yellow-orange and gets clear. The solution was allowed to stir for 15 min to assure complete reaction, with subsequent removal of the solvent under reduced pressure. The residue was washed three times with cold diethylether to obtain [((^{Ad,Me}ArO)$_3$tacn)U≡S···K(db-18c6)] as orange solid. Crystals suitable for X-ray diffraction were obtained by diffusion of diethylether into a concentrated solution of [((^{Ad,Me}ArO)$_3$tacn)U≡S···K(db-18-c-6)] in benzene.

Yield: 54 mg (n = 0.035 mmol, 87 %).

^{1}**H NMR** (400 MHz, pyridine-d_5): δ [ppm] = 106.66 (s, 3H), 53.41 (s, 3H), 51.32 (s, 3H), 9.04 (s, 4H, db18c6), 8.41 (s, 8H, db18c6), 7.83 (s, 4H, db18c6), 7.32 (s, 8H, db18c6), 3.76 (s, 3H), –0.66 (d, J = 8.07 Hz, 9H), –1.24 (d, J = 8.68 Hz, 9H), –1.59 (s, 9H), –5.59 (s, 3H), –5.67 (s, 3H), –6.59 (s, 9H), -7.98 (s, 3H), –12.43 (s, 9H), –32.18 (s, 3H), –117.54 (s, 3H).

IR (KBr pellet): $\tilde{\nu}$ (cm^{-1}) = 2901 (vs), 2845 (vs), 1732 (m), 1596 (m), 1504 (s), 1462 (vs), 1458 (vs), 1438 (m), 1417 (m), 1344 (w), 1307 (m), 1281 (m), 1254 (vs), 1221 (m), 1184 (w), 1165 (w), 1128 (s), 1107 (m) 1062 (m), 1012 (w), 998 (w), 973 (w), 956 (w), 943 (m), 892 (w), 860 (m), 826 (s), 810 (s), 778 (m), 763 (w), 752 (m), 741 (m), 678 (m), 603 (w), 573 (w), 517 (s), 414 (m), 383 (m).

Elemental analysis (%) calcd [((^{Ad,Me}ArO)$_3$tacn)U≡S···K(db-18-c-6)]: C 61.64, H 6.60, N 2.70, S 2.06; found C 61.95, H 6.33, N 2.69, S 1.51.

Synthesis of [K(2.2.2-crypt)][((^{Ad,Me}ArO)$_3$tacn)U≡S] (4). A vial was charged with [((^{Ad,Me}ArO)$_3$tacn)U-SH] (98 mg, n = 0.08 mmol) and 4 mL of THF, resulting a cloudy cyan solution. First, 2.2.2-cryptand (30 mg, n = 0.08 mmol) was dissolved in 1 mL THF and added to the reaction solution, followed by the dropwise addition of K(N(SiMe$_3$)$_2$) (16 mg, n = 0.08 mmol) in 1 mL THF. The solution turns bright orange and after 15 min an orange solid precipitates out of solution. The reaction mixture was allowed to stir for another 15 min to assure complete reaction, and the solid was collected by filtration, washed three times with 1 mL of THF, and dried *in vacuo* to obtain [K(2.2.2-crypt)][((^{Ad,Me}ArO)$_3$tacn)U≡S] as orange solid. Crystals suitable for X-ray diffraction were obtained by diffusion of toluene into a concentrated solution of [K(2.2.2-crypt)][((^{Ad,Me}ArO)$_3$tacn)U≡S] in pyridine.

Yield: 99 mg (n = 0.06 mmol, 75 %).

^{1}H NMR (400 MHz, pyridine-d_5): δ [ppm] = 106.90 (s, 3H), 53.64 (s, 3H), 51.62 (s, 3H), 4.06 (s, 12 H, Crypt), 3.89 (s, 12H, Crypt), 2.79 (s, 12H, Crypt), 0.52 (s, 3H), –0.53 (d, *J* = 7.44 Hz, 9H), –1.09 (d, *J* = 7.45 Hz, 9H), –1.52 (s, 9H), –5.28 (s, 3H), –5.58 (s, 3H), –6.66 (s, 9H), –8.10 (s, 3H), –12.60 (s, 9H), -32.58 (s, 9H), –116.88 (s, 3H).

IR (KBr pellet): $\tilde{\nu}$ (cm^{-1}) = 2897 (vs), 2842 (vs), 1602 (m), 1460 (vs), 1437 (s), 1417 (m), 1354 (m), 1341 (m), 1308 (s), 1294 (s), 1286 (s), 1265 (vs), 1225 (m), 1183 (w), 1166 (m), 1133 (s), 1106 (vs), 10749 (s), 1031 (w), 1011 (m), 975 (w), 950 (s), 932 (m), 891 (w), 861 (m), 827 (s), 810 (s), 779 (m), 764 (w), 753 (m), 573 (w), 511(s), 413 (w), 381 (m).

Elemental analysis (%) calcd [K(2.2.2-crypt)][((^{Ad,Me}ArO)$_3$tacn)U≡S]: C 59.48, H 7.30, N 4.45, S 2.04; found C 59.01, H 7.08, N 4.58, S 1.81.

Crystallographic Details

X-ray Crystal Structure Determination Details

CCDC-1452972 (**2**), CCDC-1452973 (**3**), and CCDC-1452974 (**4**) contain the supplementary crystallographic data for this paper. The data can be obtained free of charge from Cambridge Crystallographic Data Centre, 12 Union Road, Cambridge, CB2 1EZ, UK (fax: ++44-1223-336-033; e-mail: deposit@ccdc.cam.ac.uk).

Suitable single crystals of the investigated compounds were embedded in protective perfluoropolyalkyether oil on a microscope slide and a single specimen was selected and subsequently transferred to the cold nitrogen gas stream of the diffractometer. Intensity data were collected using MoK_α radiation on either a Bruker Smart APEX 2 diffractometer (λ = 0.71073 Å, graphite monochromator) for 2 or a Bruker Kappa APEX 2 *IμS* Duo diffractometer (λ = 0.71073 Å, QUAZAR focussing Montel optics) for **3** and **4.** Data were corrected for Lorentz and polarization effects, semiempirical absorption corrections were performed on the basis of multiple scans using *SADABS*.[4] The structures were solved by direct methods (*SHELXTL NT* 6.12)[5] and refined by full-matrix least-squares procedures on F^2 using *SHELXL* 2014/6.[6] All non-hydrogen atoms were refined with anisotropic displacement parameters. Tentative positions for the sulfur bound hydrogen atoms in the two independent complex molecules in the crystal structure of **2** were derived from a difference Fourier synthesis and were subsequently treated using a riding model. All other hydrogen atoms were placed in positions of optimized geometry. The isotropic displacement parameters of all H atoms were tied to those of the corresponding carrier atoms by a factor of either 1.2 or 1.5. Olex2 was used to prepare material for publication.[7] Crystallographic data, data collection, and structure refinement details are given in Table S1.

Compound **2** crystallized with two independent molecules of the U complex, each being situated on a crystallographic threefold axis. The asymmetric unit contained a number of disordered CH_2Cl_2 solvent molecules. Two alternative orientations were refined in the case of two of these solvent molecules resulting in site occupancies of 65(2) and 35(2) % for the atoms C100, Cl11, Cl12 and C110, Cl13, Cl14

and of 57(2) and 43(2) % for the atoms C200, Cl21, Cl22 and C210, Cl23, Cl24, respectively. A third CH_2Cl_2 solvent molecule was situated on a crystallographic threefold axis and was accordingly disordered. Here, no hydrogen atoms were included. DFIX, SAME, SIMU, and ISOR restraints were applied in the refinement of the disorder. Compound **3** crystallized with a total of 0.62 molecules of benzene and 0.38 molecules of Et_2O per formula unit. The two solvent molecules shared a common crystallographic site. Similarity restraints were applied to the anisotropic displacement ellipsoids of the disordered atoms (*i.e.* the two solvent molecules). In compound **4** both the uranium complex and the [K(2,2,2-crypt)] moiety were situated on threefold crystallographic axes.

A significant difference in the U–S bond distance of the two molecules of **2** (0.06 Å) was observed. Additionally, the anisotropic displacement parameters (a.d.p.'s) for the two independent S atoms look very different. We made a number of attempts to explain this different behavior by a slight deviation of the sulfur atoms from the crystallographic threefold rotation axis. Allowing the sulfur atoms to deviate from this special position and free refinement of all three coordinates does not change the overall picture of the a.d.p.'s, nor does it significantly change the U–S bond distances. In order to get a more isotropic behavior of sulfur atom S2, SIMU and ISOR restraints with very small e.s.d's proved to be necessary. A refinement of the site occupancy factors of the two sulfur atoms (as free variables) resulted in values of 0.324(5) for S1 and 0.338(5) for S2, which is very close to the expected value of ⅓ for the situation on a threefold axis. To conclude, the difference in the U–S bond distances is caused by packing forces. In the crystalline packing, one can observe distinct differences in the packing of the two independent molecules. The packing of the U1 molecules is characterized by an offset of two consecutive molecules with additional solvent molecules in between (see Figure S1), thus leaving the S atom as far as possible unaffected from neighbored complex or solvent molecules. The shortest S···C distance here is observed between S1 and an adjacent dichloromethane solvent (C100) at a distance of 3.933 Å. In contrast to this, consecutive U2 molecules are arranged in a head-to-tail fashion with no offset and without having additional solvent molecules in between. The S2 atoms are pointing to the tacn moiety of the next U2 molecule with short distances of 2.75 Å between sulfur and the tacn hydrogen atoms at C21A, C21B and C21C (distance S2···C21A is 3.716 Å). The S–H···H21X distance amounts to 1.89 Å (see Fig S2). There is obviously some steric pressure exerted on the U2–S2 bond by this short intermolecular distances.

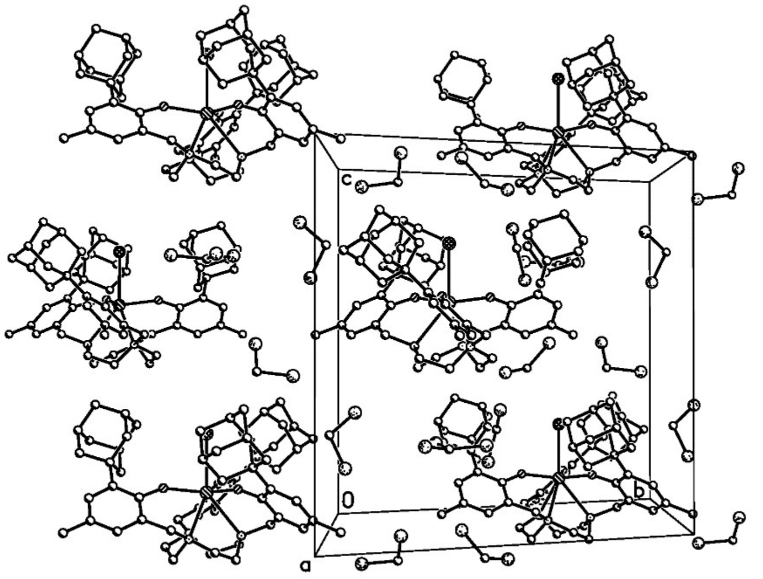

Figure S1: Packing diagram of **2** showing only the independent complex molecules at U1 and the solvent molecules of major occupancy (U2 molecules, hydrogen atoms and minor occupied solvent molecules omitted for clarity).

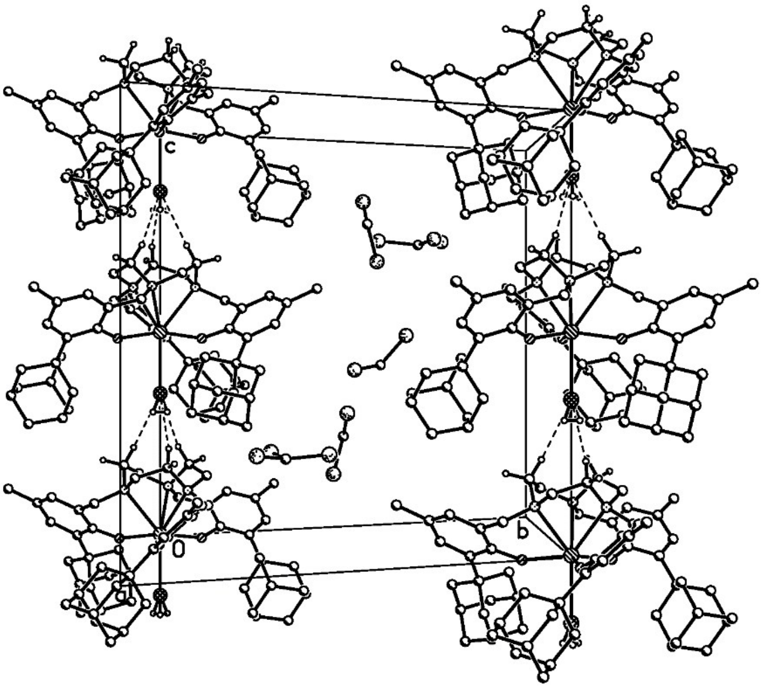

Figure S2: Packing diagram of **2** showing only the independent complex molecules at U2 and the solvent molecules of major occupancy (U1 molecules, hydrogen atoms and minor occupied solvent molecules omitted for clarity). The dotted lines indicate short contacts between sulfur and the C21x atoms of the next complex molecule.

A striking difference in U–S bond length in **3** and **4** was observed. There is without any doubt a S···K bonding interaction in **3** (the observed S···K distance of 3.136(2) Å is significantly shorter than the sum of the corresponding van der Waals radii of 4.60 Å, the sum of covalent radii is 3.07 Å). This S···K bond is on the short side of bonding distances for bonding S···K(db18-c-6) interactions (a CSD search revealed a range from 3.051 - 3.700 Å for this type of bond/interaction), which indicates a fairly strong bond in **3**. This, in turn, brings the whole potassium diphenyl-18-crown-6 moiety close to the uranium complex. Its steric influence is visible by the significant differences between the U–N_{tacn} bonds. One short U–N bond of 2.704(3) Å (U1–N1) is observed at the side of the $(^{Ad,Me}ArO)_3$tacn ligand, where the potassium diphenyl-18-crown-6 moiety enters the uranium complex' periphery. The other two U–N_{tacn} bonds on the opposite side amount to 2.883(3) Å for U1–N2 and 2.870(3) Å for U1–N3. In a similar way, the ArO ring on the cation's side (O1, C8 - C13) is pushed away from the potassium diphenyl-18-crown-6 moiety, as can be seen from the torsion angle U1–O1–C9–C8 (-17.3(6)°), while the corresponding torsion angles on the other side of the uranium complex are significantly larger by more than 5° (U1–O2–C27–C26: 25.8(7)° and U1–O3–C45–C44: 22.2(8)°). Last but not least, the U center in **3** lies to a minor amount below the plane formed by the three oxygen donor atoms of the $(^{Ad,Me}ArO)_3$tacn ligand than in **4** (out-of-plane shifts are –0.055 Å in **3** and –0.086 Å in **4**). Therefore, the striking differences observed for the U–S bond distances in **3** and **4** might again be explained by the steric situation in the two complexes. In compound **4**, the sulfur atom is only bonded to the uranium center without further steric constraints. In **4**, however, the sulfur atom is bonded both to the uranium center of the complex and to the potassium of the complex cation. In this situation one would, at first sight, expect an elongation of the U–S bond but a shortening (compared to the observed U–S bond in **4**) is observed. The dibenzo-18-crown-6 moiety exerts a considerable steric strain that might push the sulfur atom slightly deeper into the cavity of the

[((^{Ad,Me}ArO)$_3$tacn)U] moiety, while at the same time the uranium reduces its negative out-of-plane shift and moves closer to the sulfur atom, in order to accommodate the sterically demanding potassium dibenzo-18-crown-6 moiety in the complex periphery. As we could already see from the differences in the U–S bond distances in the two independent molecules in **2**, it can be attributed to the packing forces and, therefore, the steric situation that causes significant changes in the length of the observed U–S bond.

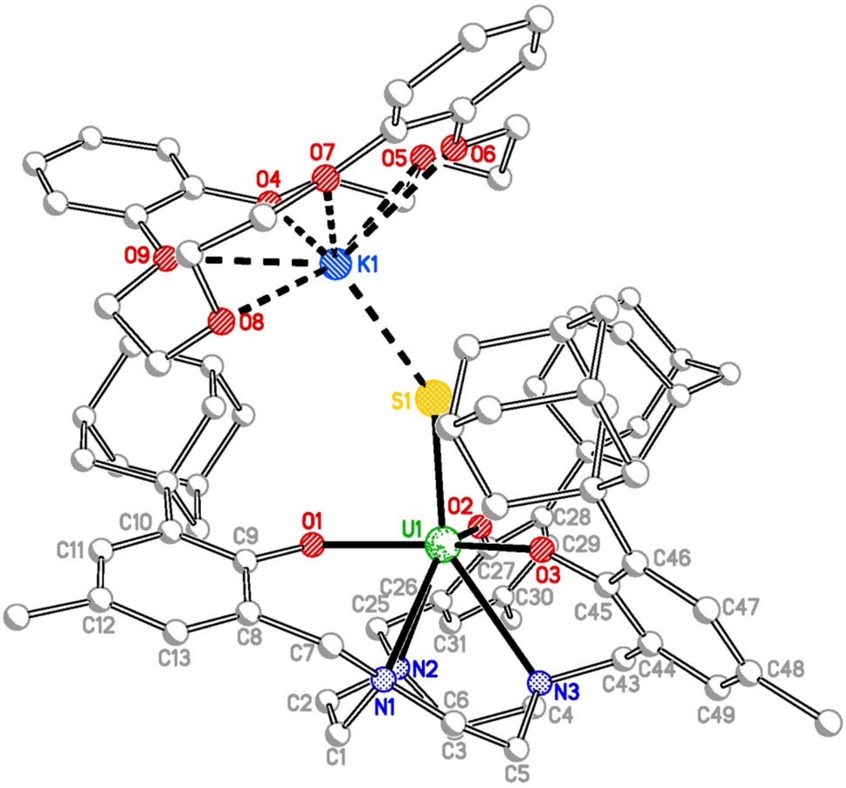

Figure S3: Ball-and-stick representation of the molecular structure of **3** highlighting the steric pressure exerted by the potassium diphenyl-18-crown-6 moiety on the left side of the complex molecule (hydrogen atoms and solvent molecules omitted for clarity).

Table S1: Crystallographic data, data collection, and refinement details for **2**, **3**, and **4**.

	**2**	**3**	**4**
Empirical formula	$C_{63.25}H_{85.}5Cl_{6.53}N_3O_3SU$	$C_{85.23}H_{109.53}KN_3O_{9.38}SU$	$C_{78}H_{114}KN_5O_9SU$
Mol. Weight	1436.36	1635.38	1574.93
Crystal shape, color	prism, light green	block, orange	hexagonal prism, orange
Crystal size [mm]	0.22 x 0.11 x 0.1	0.22 x 0.13 x 0.08	0.07 x 0.06 x 0.04
Temperature [K]	100	100	100
Crystal system	hexagonal	monoclinic	hexagonal
Space group	$P6_3$	$P2_1/c$	$P6_3$
a [Å]	20.258(5)	21.017(2)	14.901(2)

b [Å]	20.258(5)	12.856(2)	14.901(2)
c [Å]	18.302(4)	28.173(3)	19.249(3)
α [°]	90	90	90
β [°]	90	101.271(3)	90
γ [°]	120	90	120
V [Å^3]	6505(3)	7465(2)	3701(2)
Z	4	4	2
ρ [g cm^{-3}] (calc.)	1.467	1.455	1.413
μ [mm^{-1}]	2.839	2.320	2.337
F (000)	2914	3376	1632
T_{min};T_{max}	0.646; 0.746	0.672; 0.746	0.624; 0.746
2Θ interval [°]	2.3 ≤ 2 Θ ≤ 57.5	1.9 ≤ 2 Θ ≤ 55.9	3.1 ≤ 2 Θ ≤ 55.8
Coll. Refl.	165921	233649	71363
Indep. Refl.; R_{int}	11217	17883	5903
Obs. refl. F_0 ≥4σ(F)	10199	15242	4066
No. ref. param.	532	949	287
wR_2 (all data)	0.0714	0.0867	0.0645
R_1 (F_0 ≥4σ(F))	0.0307	0.0434	0.0319
GooF on F^2	1.160	1.287	1.028
$\Delta\rho_{max/min}$ [e Å^{-3}]	1.15; –0.80	1.302; –0.973	0.721; –0.626
Absolute structure parameter[8]	0.015(7)	—	0.002(5)

Graphical representations

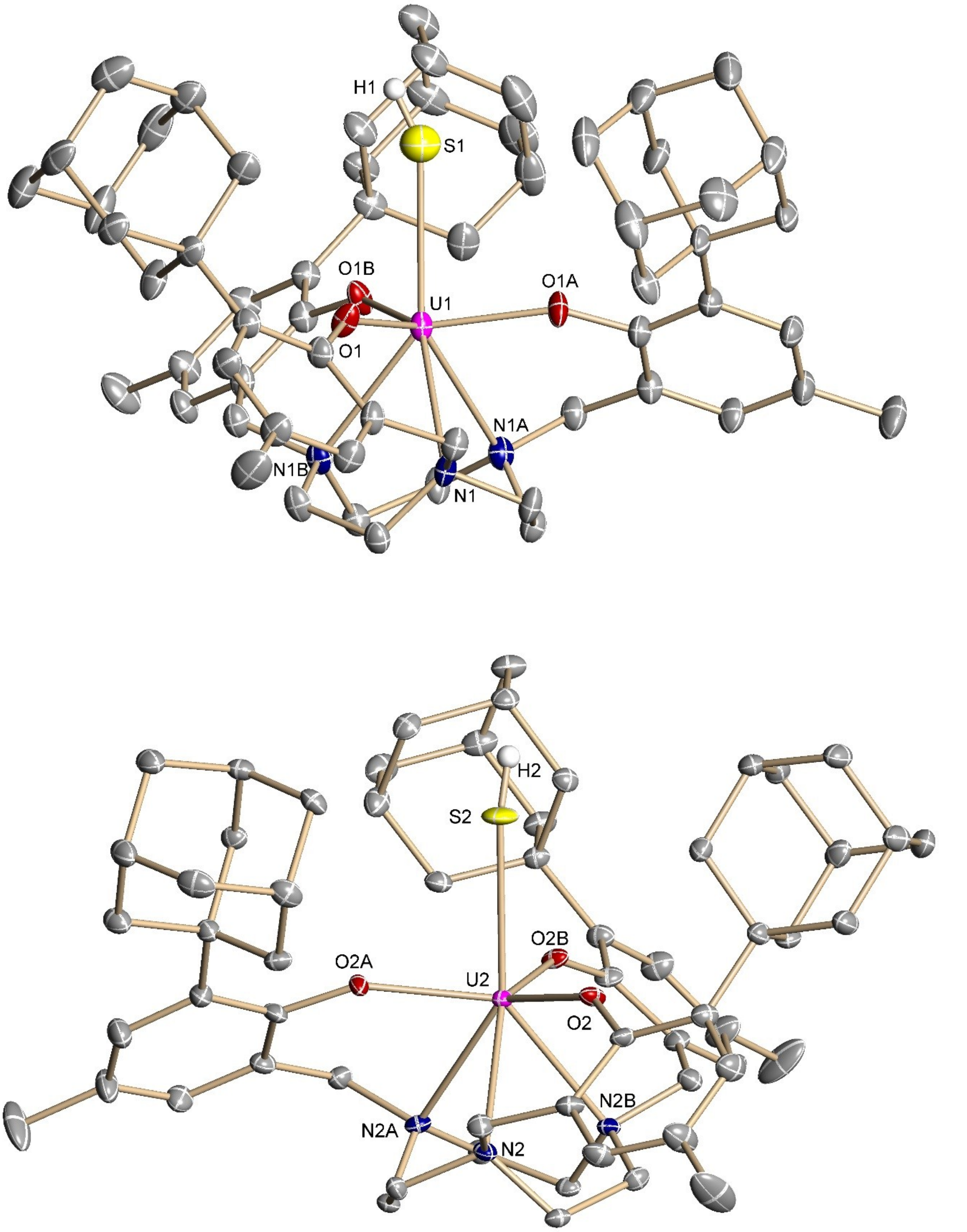

Figure S4: Molecular structures of the two crystallographically independent molecules per asymmetric unit in crystals of **2** · 3.25 CH_2Cl_2. Non-sulfur bound hydrogen atoms and co-crystallized solvent molecules are omitted for clarity. Thermal ellipsoids are drawn at 50% probability.

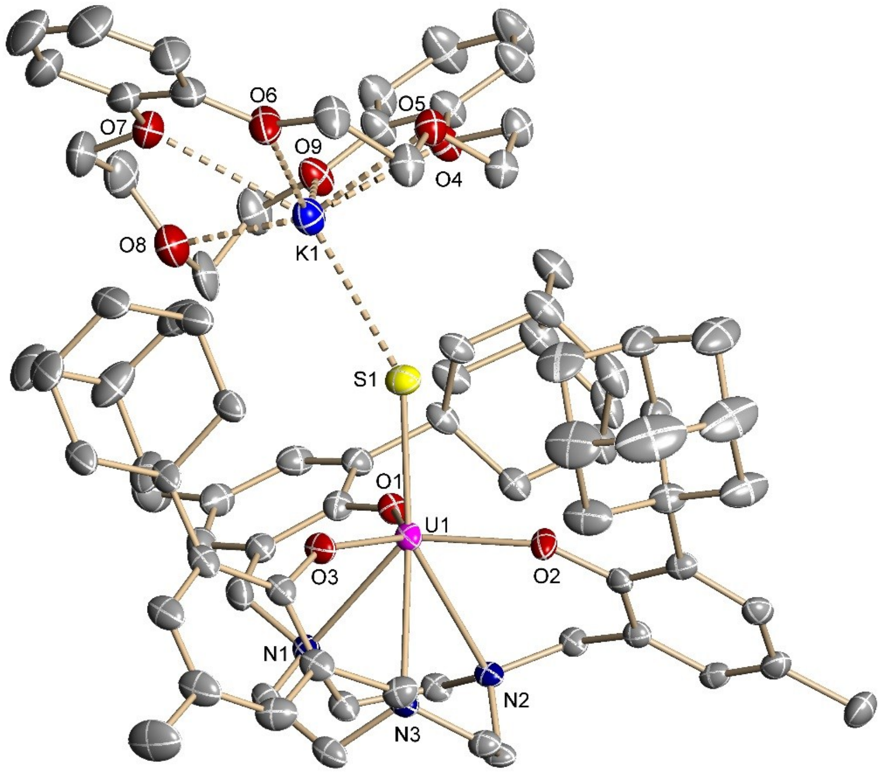

Figure S5: Molecular structure of **3** in crystals of **3** · 0.62 benzene · 0.38 Et_2O. Hydrogen atoms and co–crystallized solvent molecules are omitted for clarity. Thermal ellipsoids are drawn at 50% probability.

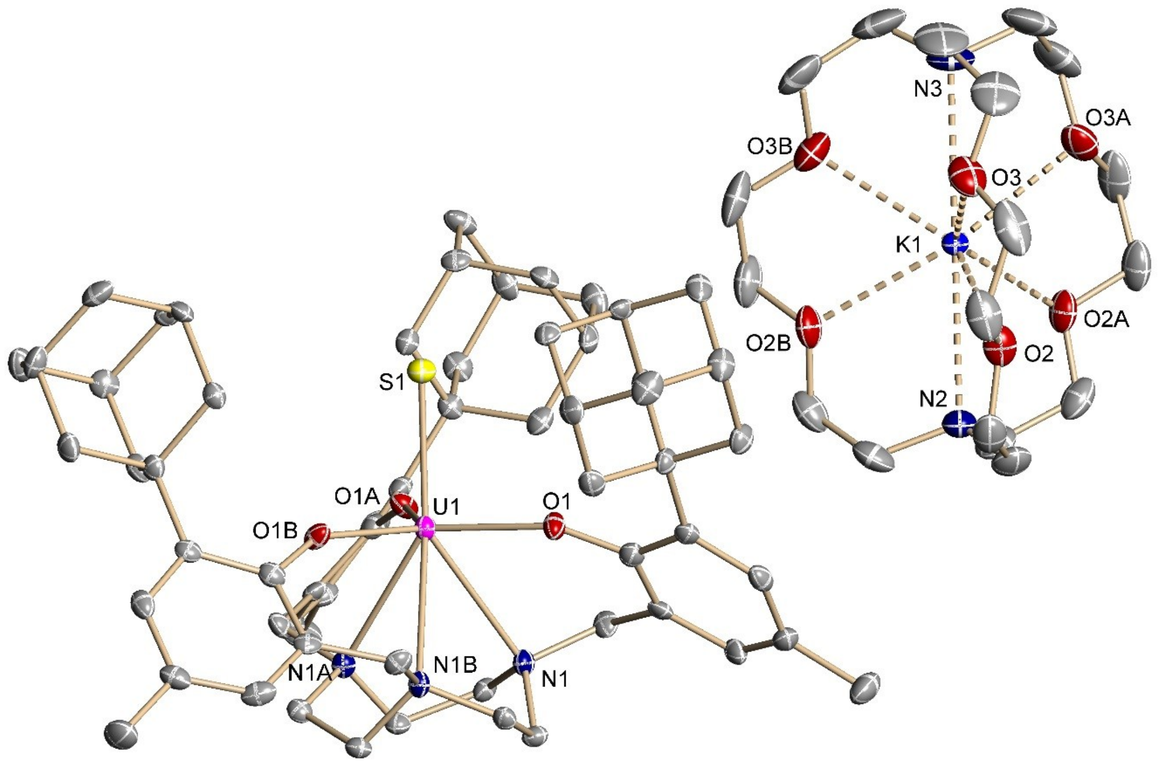

Figure S6: Molecular structure of **4**. Hydrogen atoms are omitted for clarity. Thermal ellipsoids are drawn at 50% probability.

^{1}H NMR spectroscopy

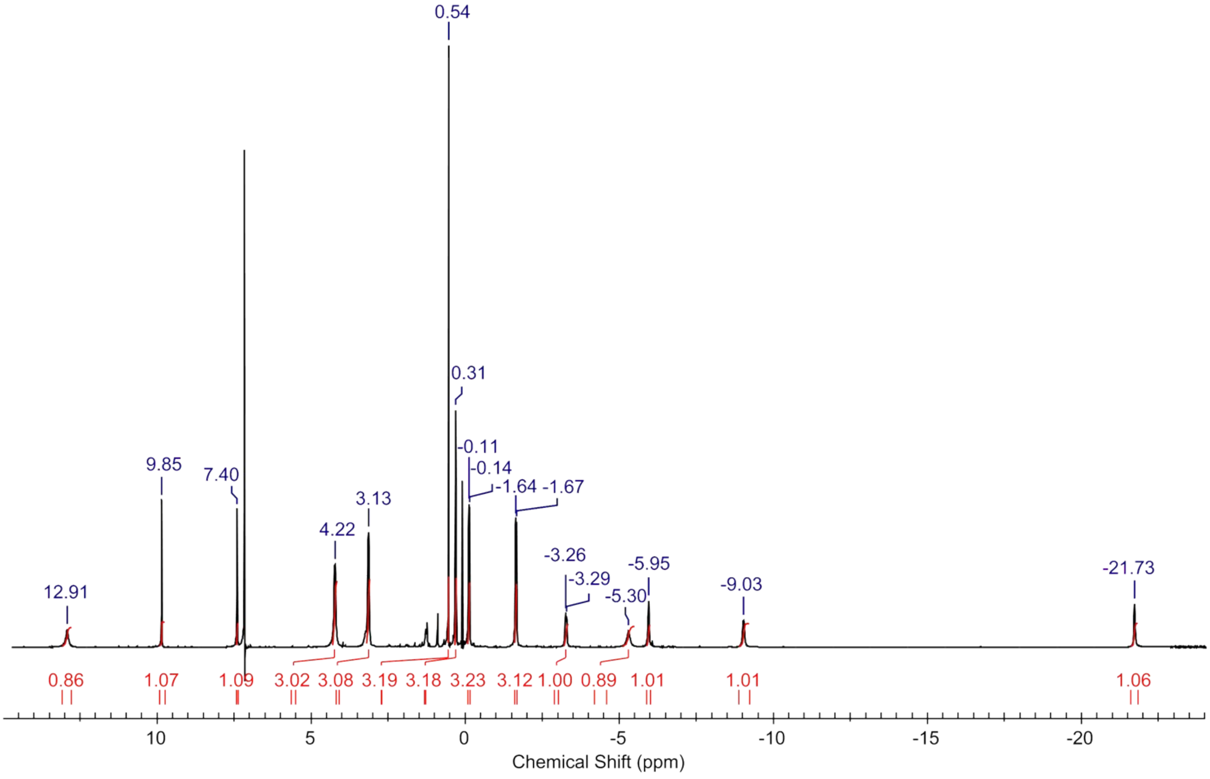

Figure S7: ^{1}H NMR spectrum of **1** recorded in benzene-d_6.

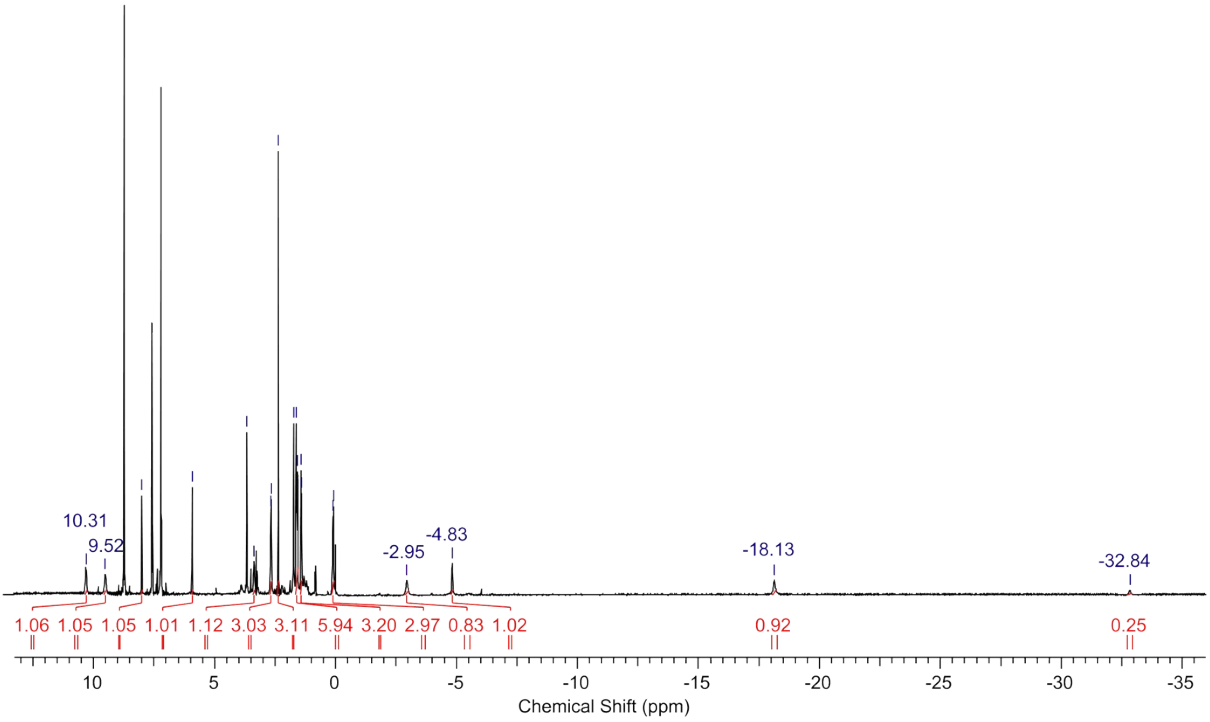

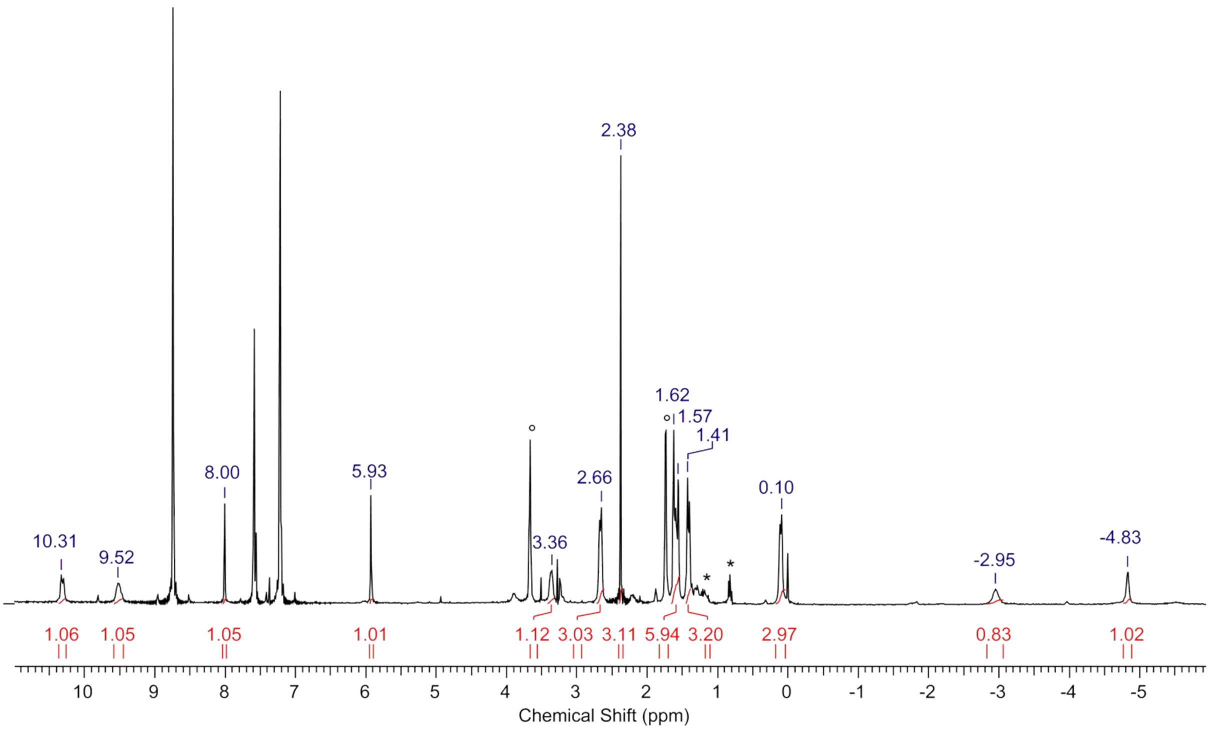

Figure S8: ^{1}H NMR spectrum of **2** recorded in pyridine-d_5. Full range spectrum (top) and zoomed in part (bottom). The spectrum still shows some solvent impurities (* Pentane, ° THF).

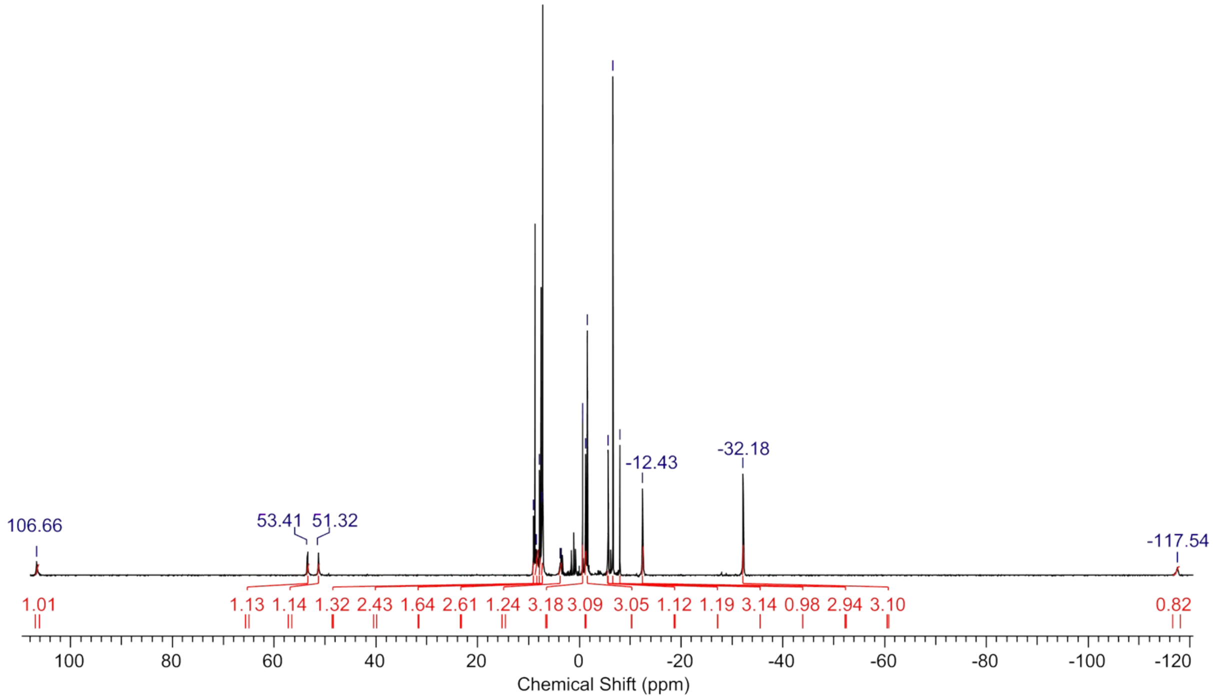

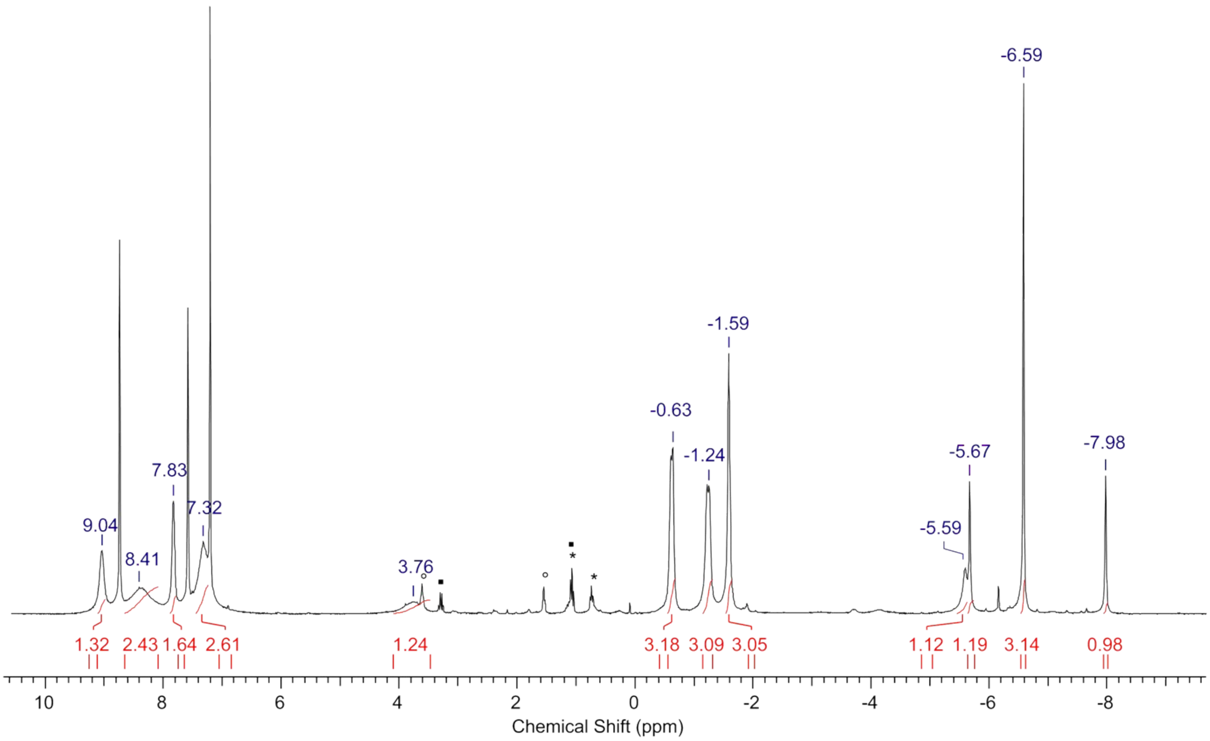

Figure S9: ^{1}H NMR spectrum of **3** recorded in pyridine-d_5. Full range spectrum (top) and zoomed in part (bottom). The spectrum still shows some solvent impurities (* Pentane, ▪ diethyl ether, ° THF).

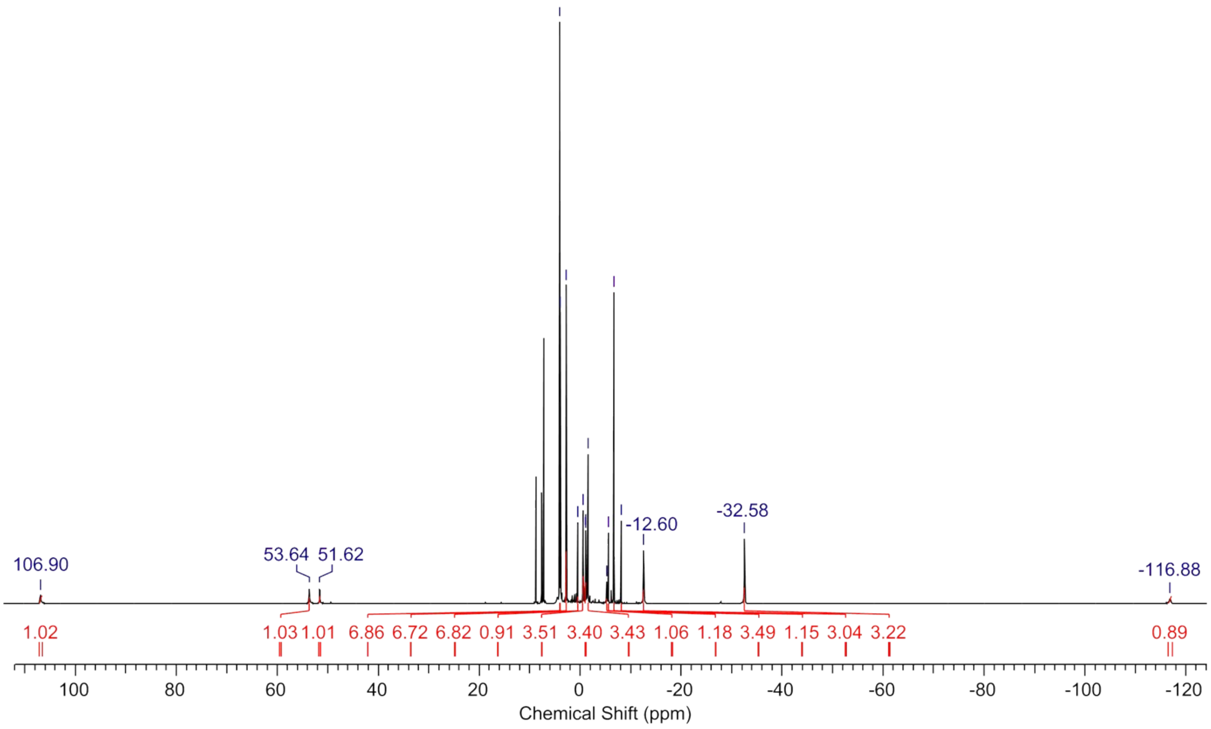

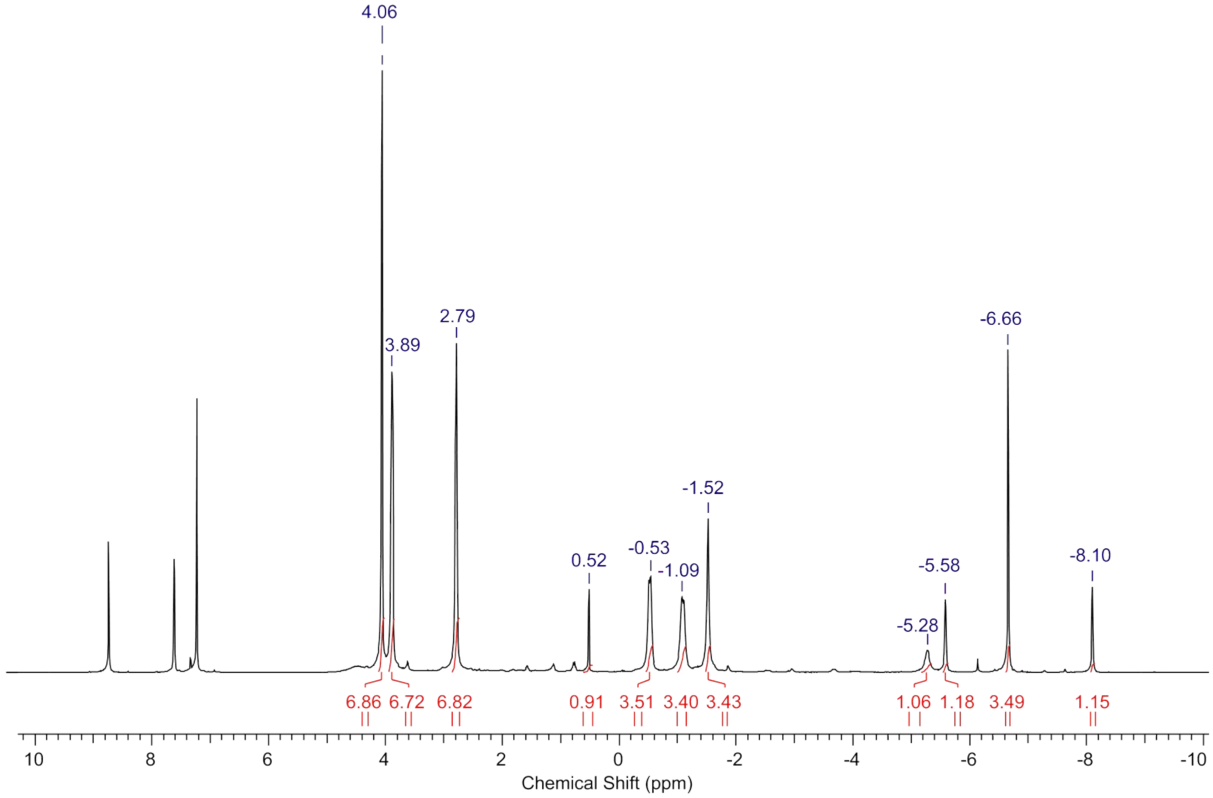

Figure S10: ^{1}H NMR spectrum of **4** recorded in pyridine-d_5. Full range spectrum (top) and zoomed in part (bottom).

IR spectroscopy

In order to get insight into the strength of the uranium–sulfido bond, infrared vibrational spectroscopy was performed on complexes **2**–**4**. Additionally, IR spectroscopy was expected to be a useful tool to prove the presence of the SH species, because the weak Î$_{\text{–(SH)}}$ band should be observable between 2600–2300 cm^{-1}, a region were typically no other absorptions are observed. However, the Î$_{\text{–(SH)}}$ band is not detectable for hydrosulfido complex **2**, an observation that was previously also made for other transition metal complexes[9-15] as well as the only other structurally characterized uranium hydrosulfido complex known, $[((^{Ad,Me}ArO)_3N)U–SH]$.[16] Since the U≡S absorption frequency of the terminal sulfido species would be a direct experimental measure of the uranium sulfido bond strength, IR spectroscopy was also performed on complexes **3** and **4**. However, the fingerprint region of both terminal sulfido compounds is identical, with the exception of absorption bands resulting from db-18-c-6 or 2.2.2-crypt, implying that the U≡S absorption band lies either below 400 cm^{-1} or is superimposed by ligand absorption bands.

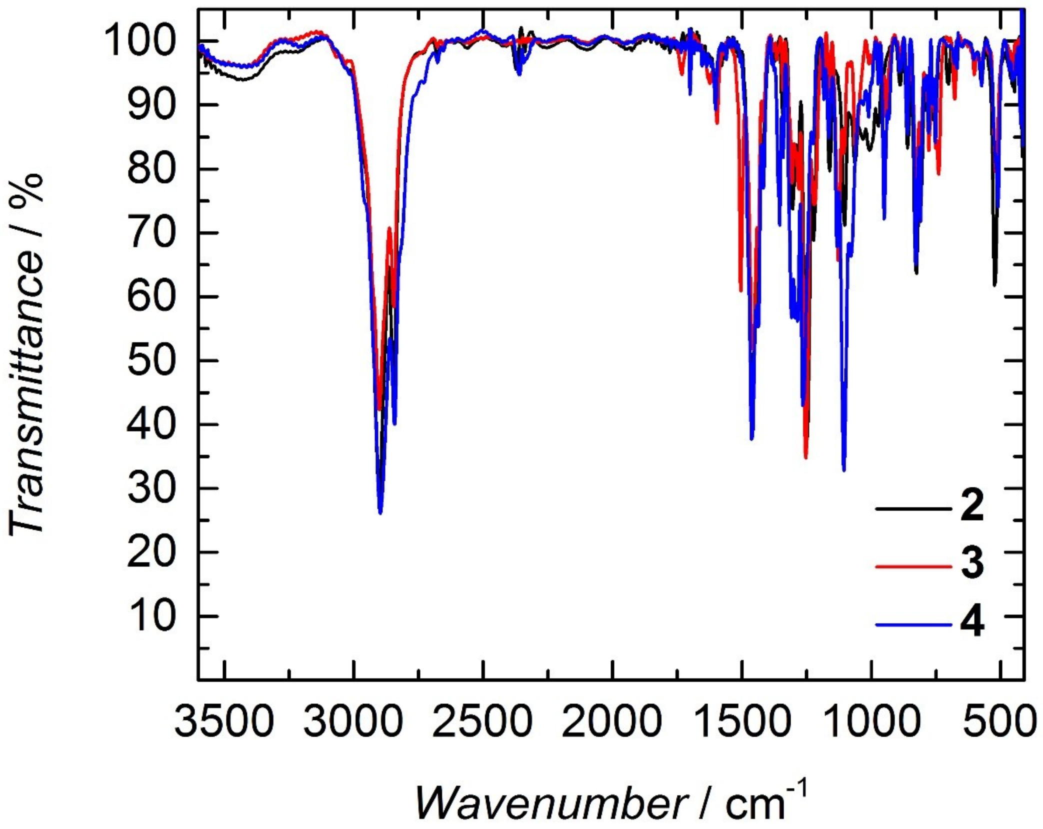

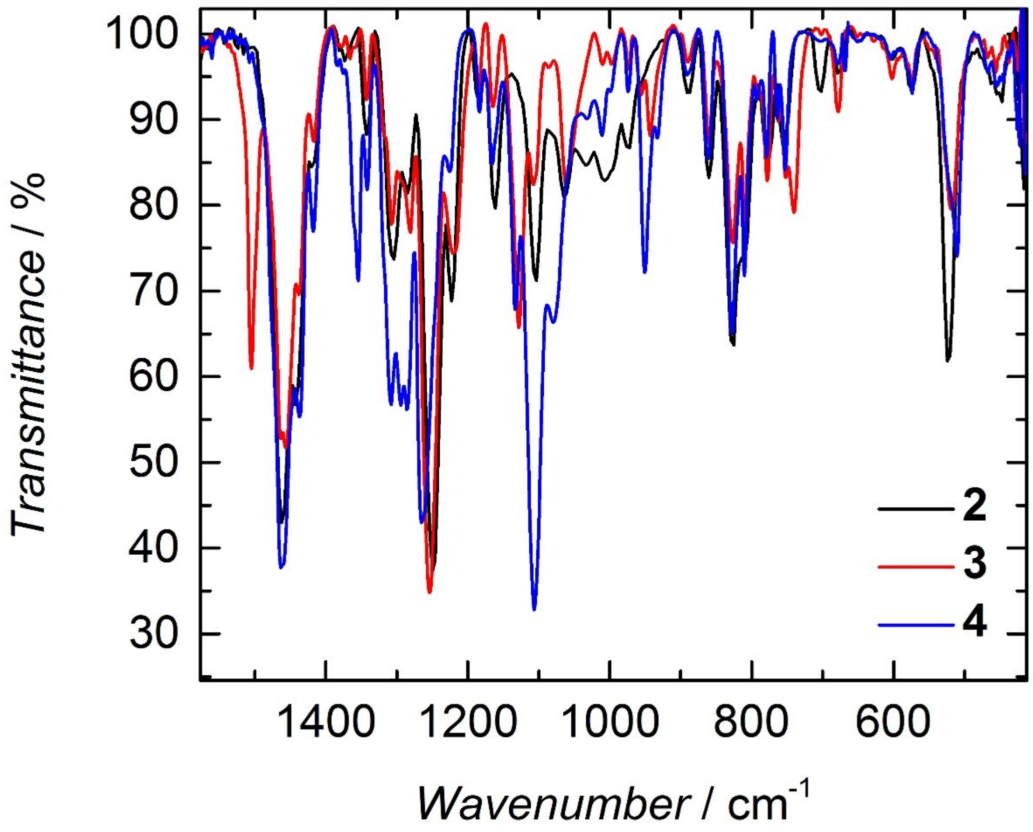

Figure S11: IR spectra of [((^{Ad}ArO)$_3$tacn)U–SH] **2** (black), [((^{Ad,Me}ArO)$_3$tacn)U≡S···K(db-18-c-6)] **3** (red), and [K(2.2.2-crypt)][((^{Ad,Me}ArO)$_3$tacn)U≡S] **4** (blue). Full range spectrum (top) and zoomed in part (bottom).

Electronic Absorption Spectroscopy

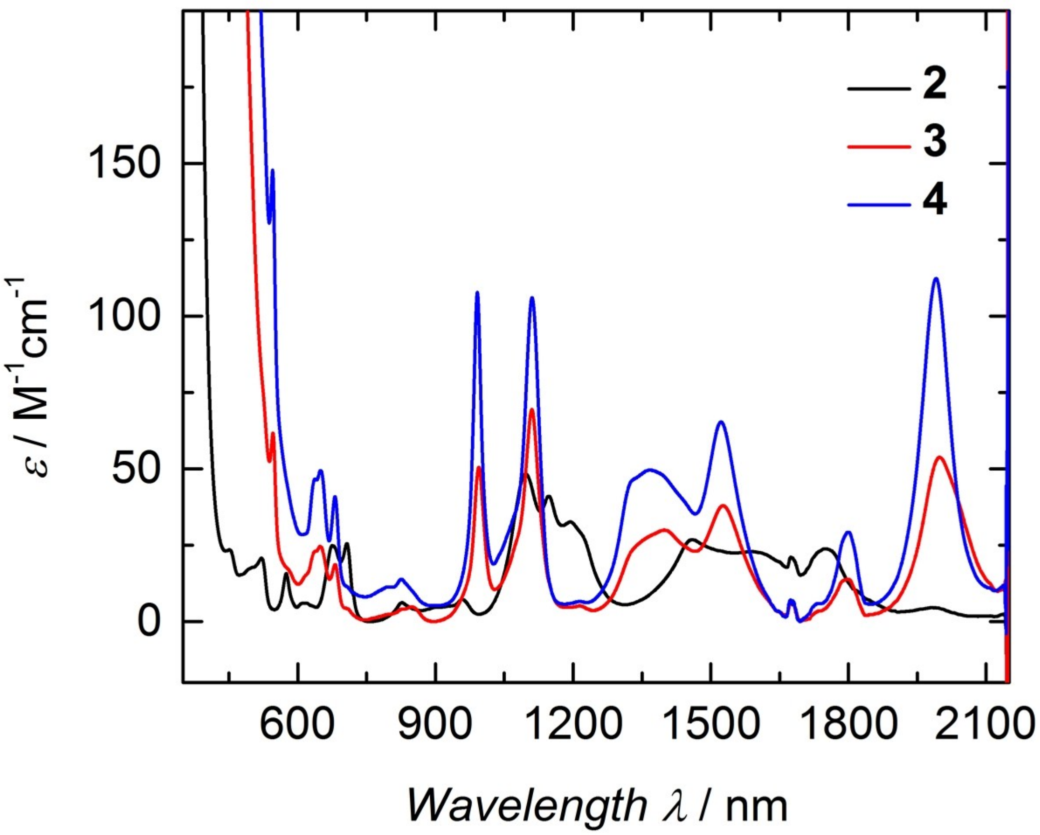

Figure S12: Vis/NIR spectra of complexes [((^{Ad}ArO)$_3$tacn)U–SH] (**2,** black), [((^{Ad,Me}ArO)$_3$tacn)U≡S···K(db-18-c-6)] (**3**, red), and [K(2.2.2-crypt)][((^{Ad,Me}ArO)$_3$tacn)U≡S] (**4**, blue). Samples were measured as 5 mM solutions in pyridine.

Table S2: List of absorption bands and related extinction coefficients for complexes **2** – **4** in the vis/NIR region.

**band #**	**2**	**3**	**4**
	**λ (nm) / ε (M^{-1}cm^{-1})**	**λ (nm) / ε (M^{-1}cm^{-1})**	**λ (nm) / ε (M^{-1}cm^{-1})**
1	1985 / 4	1998 / 54	1990 / 112
2	1750 / 24	1801 / 14	1799 / 29
3	1590 / 23	1527 / 38	1522 / 65
4	1460 / 27	1393 / 30	1367 / 50
5	1194 / 33	1111 / 69	1111 / 116
6	1147 / 41	994 / 50	991 / 108
7	1096 / 48	846 / 4	825 / 14

8	959 / 7	827 / 8	797 / 11
9	827 / 6	682 / 19	682 / 41
10	707 / 25	649 / 25	650 / 49
11	676 / 25	639 (sh) 23	639 (sh) / 47
12	615 / 6	546 / 62	545 / 148
13	575 / 16		
14	521 / 21		
15	452 / 23		

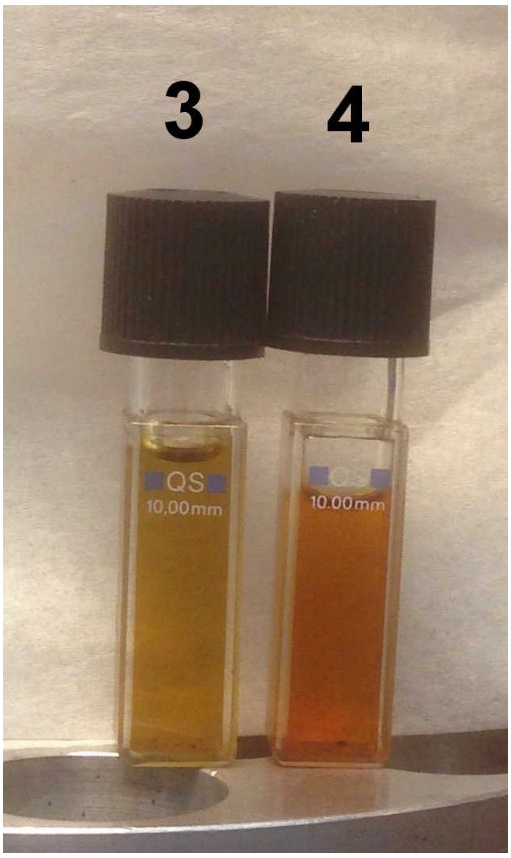

Figure S10: Picture of vis-NIR samples of **3** (*left*) and **4** (*right*) 5 mM in pyridine.

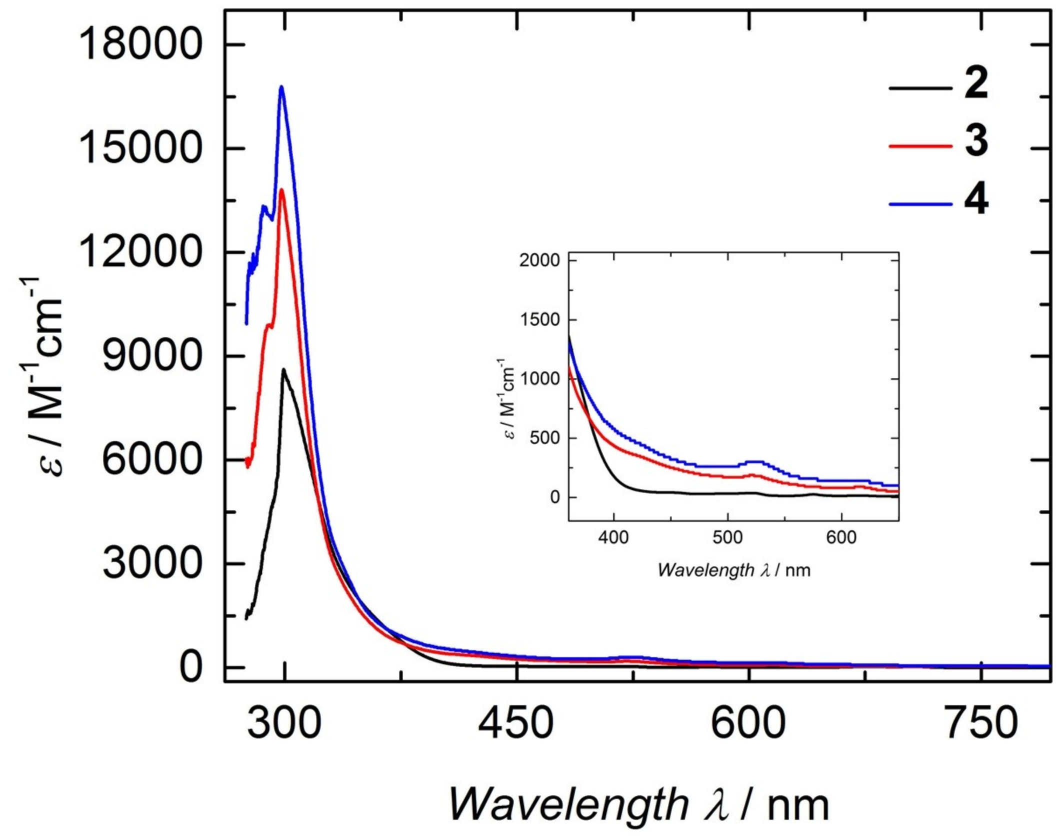

Figure S14: UV/vis spectra of complexes [((^{Ad}ArO)$_3$tacn)U–SH] (**2**, black), [((^{Ad,Me}ArO)$_3$tacn)U≡S···K(db-18-c-6)] (**3**, red), and [K(2.2.2-crypt)][((^{Ad,Me}ArO)$_3$tacn)U≡S] (**4**, blue). Samples were measured as 0.1 mM solutions in pyridine.

Table S3: List of absorption bands and related extinction coefficients for complexes **2** – **4** in the UV/vis region.

**band #**	**2**	**3**	**4**
	**λ (nm) / ε (M^{-1}cm^{-1})**	**λ (nm) / ε (M^{-1}cm^{-1})**	**λ (nm) / ε (M^{-1}cm^{-1})**
1	299 / 8622	524 / 190	524 / 300
2		298 / 13780	298 / 16800
		289 / 9880	287 / 13200

Magnetism

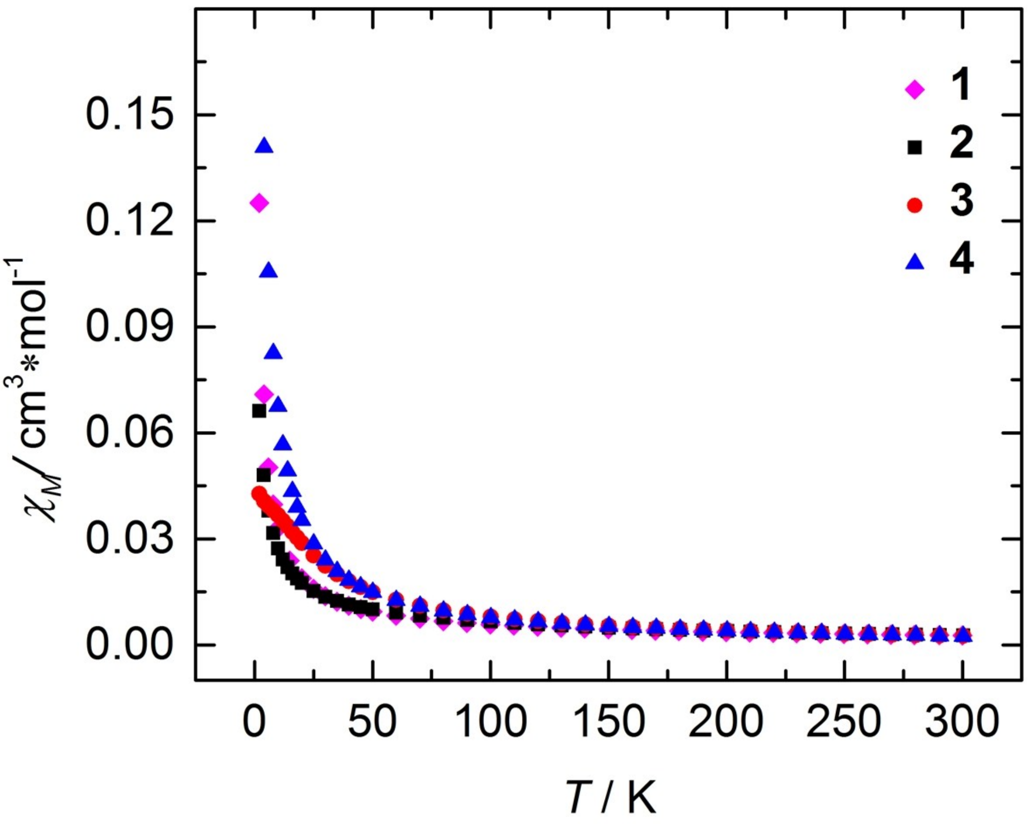

0.15
0.12
0.09
0.06
0.03
0.00
χM / cm3*mol-1
0
50
100
150
200
250
300
T / K
1
2
3
4

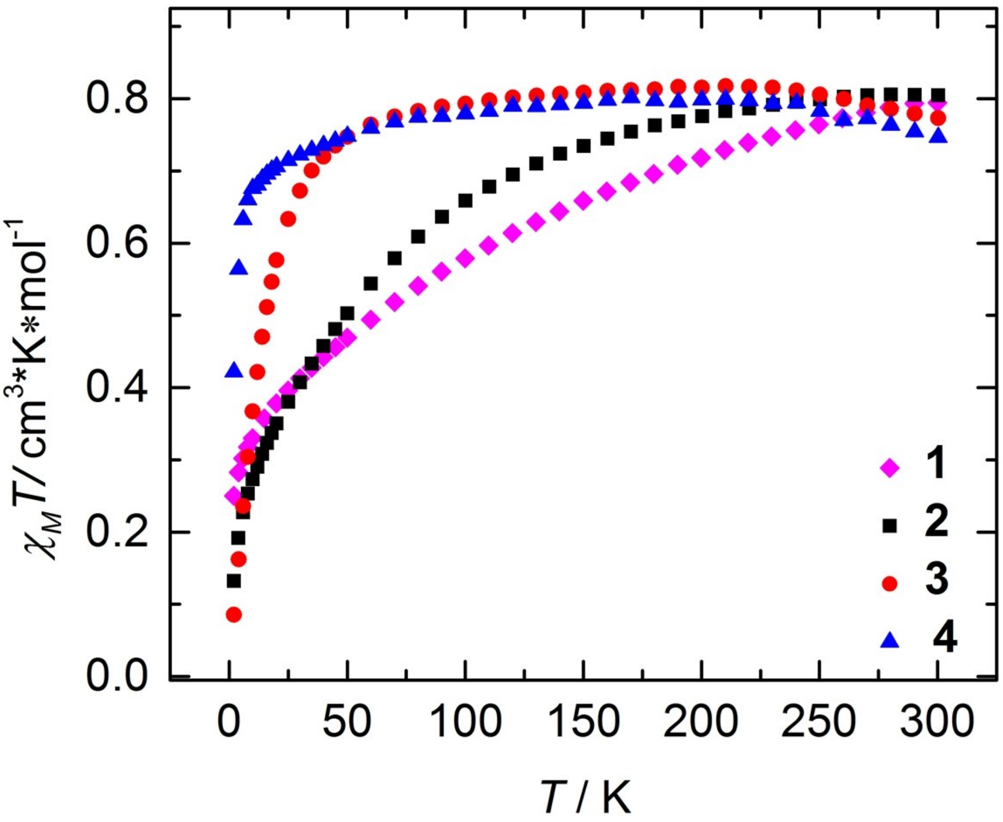

0.8
0.6
0.4
0.2
0.0
$\chi_M T$/ cm^3*K*mol^{-1}
0
50
100
150
200
250
300
T / K
1
2
3
4

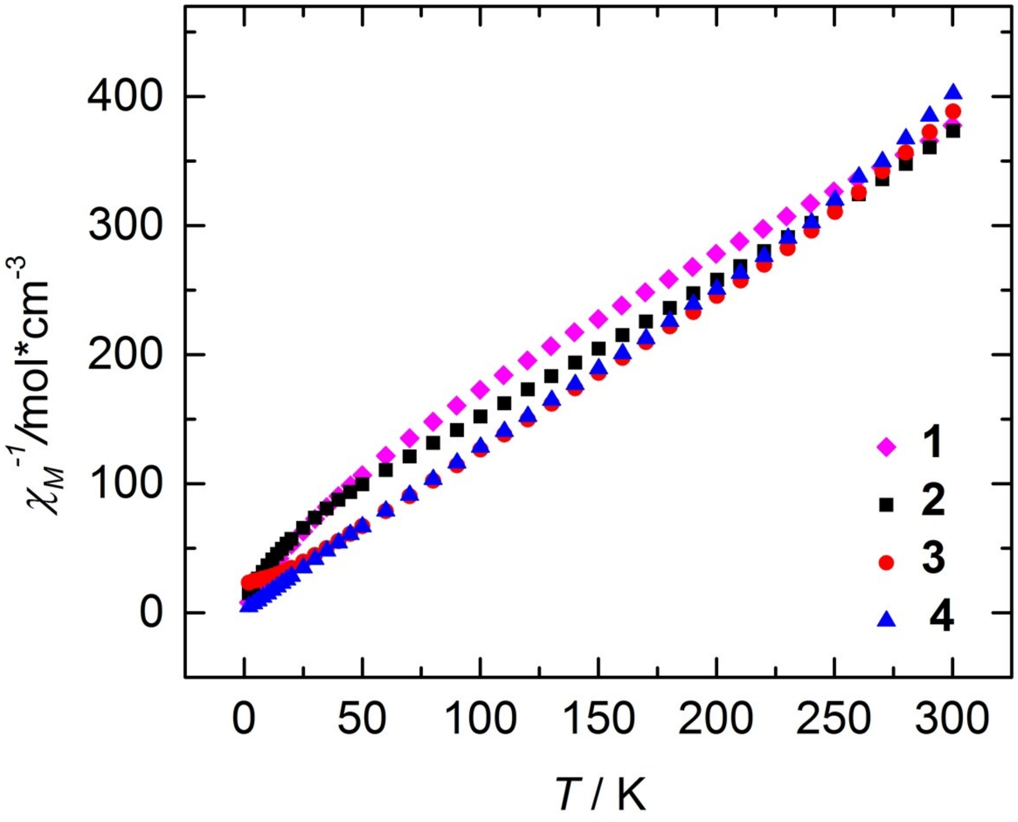

400
300
200
100
0
χ_M^{-1}/mol*cm^{-3}
0
50
100
150
200
250
300
T / K
1
2
3
4

Figure S15: Temperature-dependent SQUID magnetization data of uranium(III) complex **1** (magenta), terminal uranium(IV) hydrosulfido complex **2** (black), sulfido complexes **3** (red), and **4** (blue). Data as a plot of χ_{molar} *vs*. T, $\chi_{molar}T$ *vs*. T, and χ_{molar}^{-1} *vs*. T. Data were corrected for underlying diamagnetic susceptibility. Reproducibility was checked by at least two independently synthesized samples for each compound.

Cyclic Voltammetry

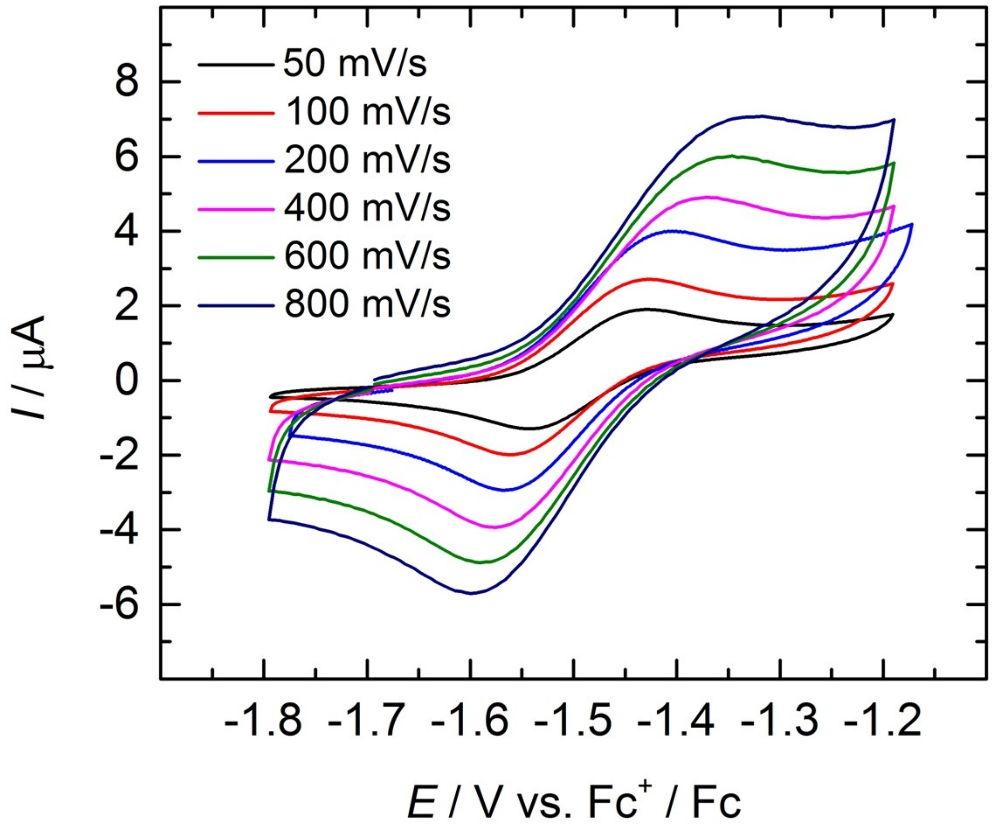

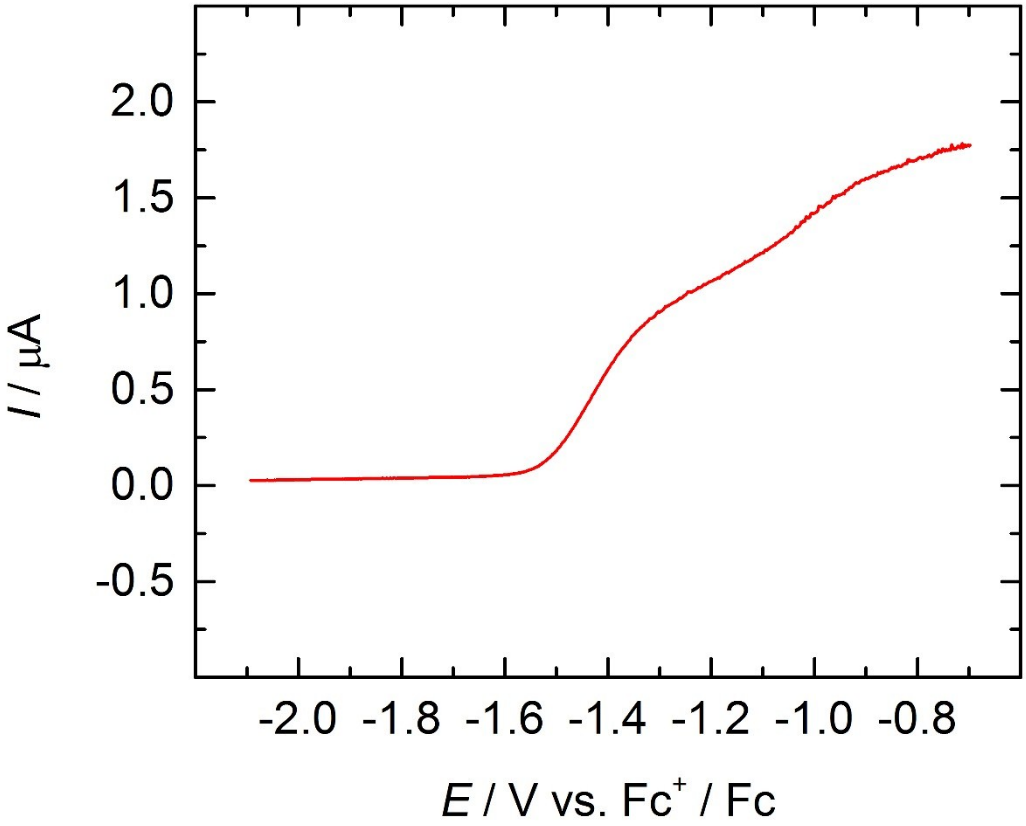

Figure S16: Cyclic voltammogram of [((^{Ad,Me}ArO)$_3$tacn)U≡S···K(db-18-c-6)] (**3**), recorded at different scan rates in ~0.1 M [N(*n*-Bu)$_4$][BPh$_4$] in THF at room temperature (top). Linear-sweep measurement of oxidation potential of **3** (bottom). The scan was collected at 50 mV/s with a step potential of 2 mV. Half-wave potential was measured –1.494 V *vs*. Fc$^+$/Fc.

Computational Study

The Gaussian09 program suite was used for performing all the quantum-chemical calculations.[17] As functional we have used the Becke's 3-parameter hybrid one,[18] combined with the non-local correlation functional provided by Perdew/Wang,[19] denoted as B3PW91. The relativistic energy-consistent small-core pseudopotential of the Stuttgart-Köln ECP library was used in combination with its adapted segmented basis set to represent uranium atom, going from +III to +IV to +V complexes.[20-22] For the potassium and sulphur, the quasi-relativistic energy-adjusted *ab-initio* pseudopotentials wasused, along with its corresponding energy-optimized valence basis sets,[23, 24] augmented by a *d* polarization function, for the case of silicon atoms.[25] For all the atoms, the 6-31G(d) basis set was used,[26, 27] In all computations no constrains were imposed on the geometry. Full geometry optimization was performed for each structure using Schlegel's analytical gradient method[28] and the attainment of the energy minimum was verified by calculating the vibrational frequencies that result in absence of imaginary eigenvalues. All stationary points have been identified for minimum (number of imaginary frequencies Nimag=0) Finally, for the 3D representation of the structures and the orbital plor the Chemcraft graphical program was used.[29]

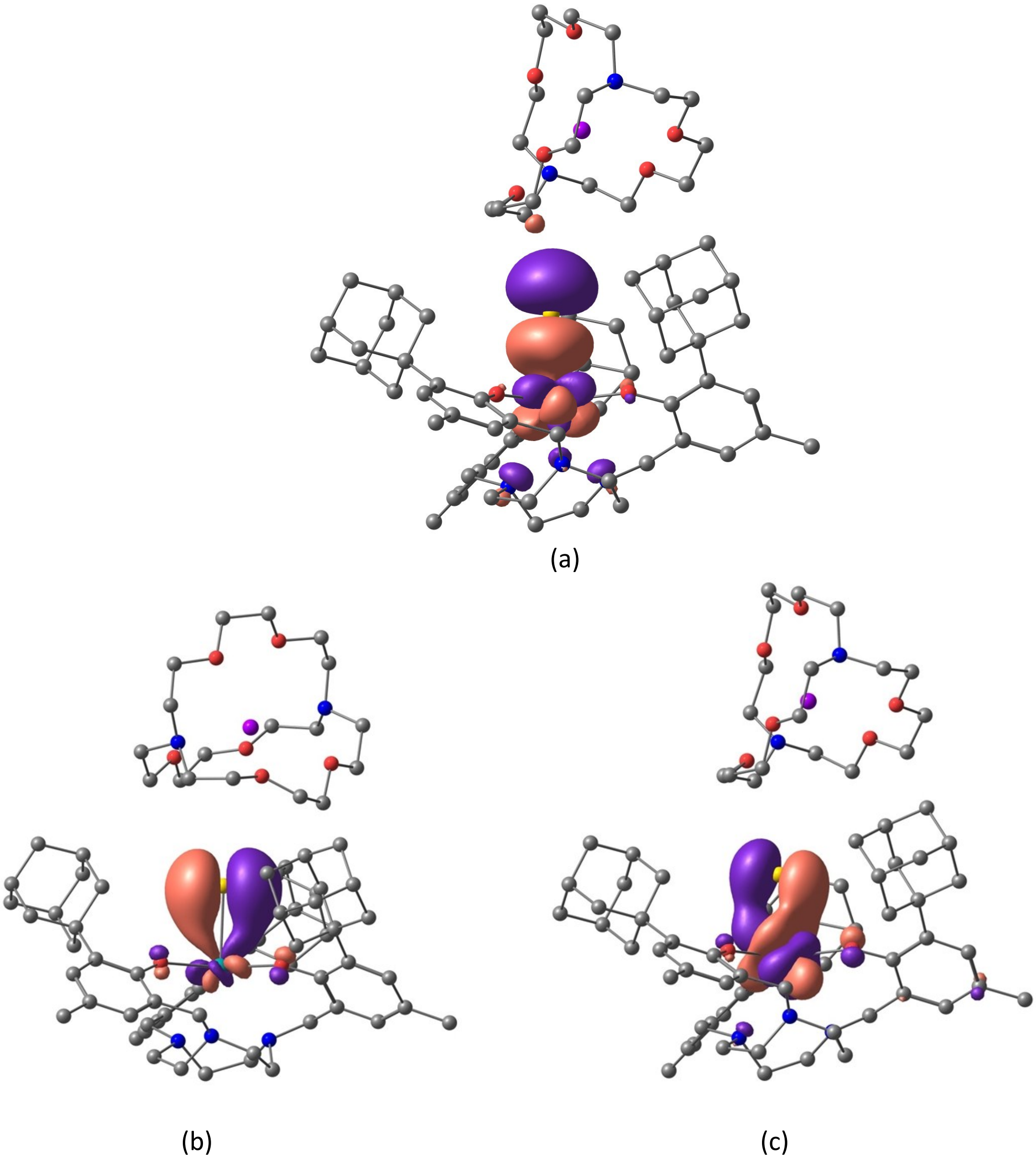

Figure S17: U—S bonding orbitals in complex **4**. (a) is a σ orbital and (b) and (c) are the two π orbitals.

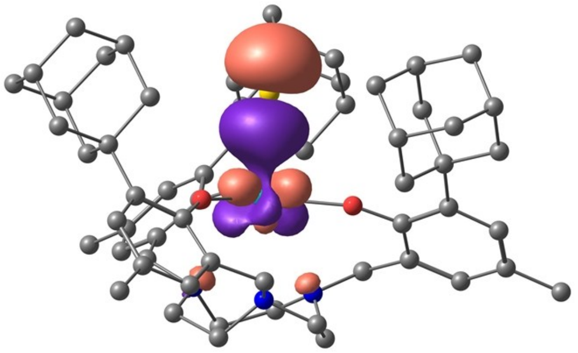

(a)

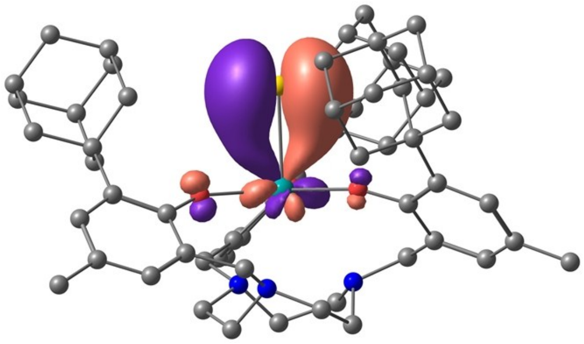

(b)

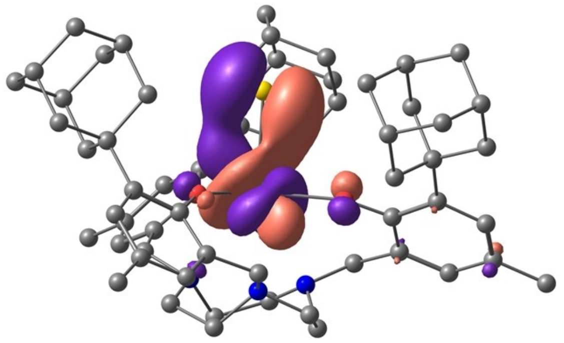

(c)

Figure S18: U—S bonding orbitals in complex **4**⁻ without counterion. (a) is a σ orbital and (b) and (c) are the two π orbitals.

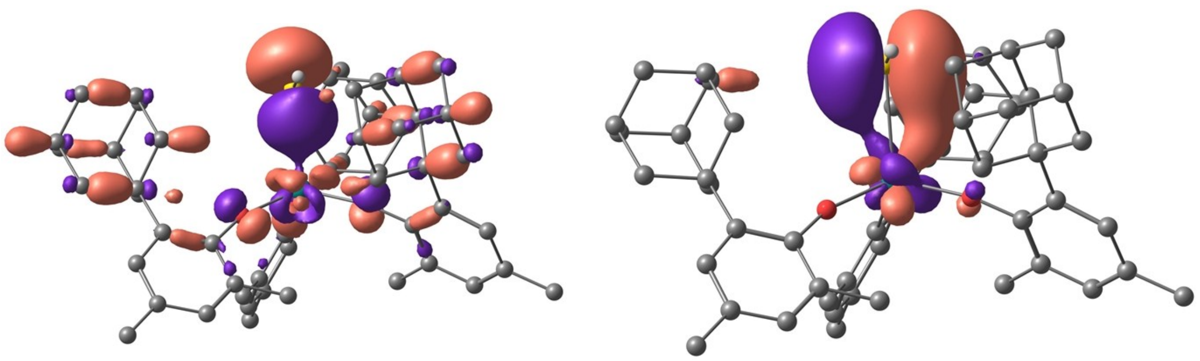

Figure S19: U—S bonding orbitals in model complex **2*** without tacn. Left is a σ orbital and right is the π orbital.

Table S4: Computed U—X bond length for complexes **2-4**, as well as for the oxygen analogs of **3** and **4**⁻.

	Bond distance in Å	
	S	O
UXH	2.83	2.14
UX (with K-crown ether)	2.65	2.03
UX	2.58	1.97
UX (with K-crypt)	2.63	2.00

	Second order NBO
UOH	62.85 (σ)
UO	87.27 (σ), 44.79 (π), 45.41 (π)

Cartesian coordinates of optimized complexes

Complex 3

197

Coordinates

U 0.094466 -2.606603 6.586233

K -2.785231 1.828136 4.854908

S -0.787061 -0.158847 6.077003

O -0.246400 -3.100791 4.396998

O 2.130065 -1.794324 7.137048

O -1.535422 -2.776453 8.153627

O -5.107936 1.210016 3.415367

O -2.996875 2.189297 1.848457

O -1.036238 3.397427 3.085883

O -1.070983 3.877128 5.859529

O -3.503936 3.498668 7.113226

O -5.497485 3.039234 5.488536

N 2.147412 -4.508982 6.070616

N 0.761950 -4.431621 8.655172

N -0.681231 -5.277537 6.228567

C 0.307690 0.209497 0.117486

C 0.359097 0.125164 1.648039

C 1.814314 -0.064359 2.102617

C 2.377457 -1.356595 1.491194

C 2.319616 -1.273127 -0.039482

C 0.858242 -1.094195 -0.476316

C -0.484433 -1.062518 2.133890

C 0.054293 -2.397126 1.558528

C 1.539597 -2.551448 1.967637

C 0.020002 -2.285408 0.017416

C -0.769035 -3.574077 2.089381

C -0.880920 -3.835659 3.490545

C -1.681671 -4.919805 3.920748

C -2.335118 -5.735751 2.993960

C -2.235057 -5.506613 1.626704

C -1.451611 -4.422116 1.214105

C 3.457704 -1.886871 7.111999

C 4.057525 -2.871535 6.293572

C 5.443944 -3.036222 6.271981

C 6.280234 -2.236264 7.041956

C 5.676341 -1.244893 7.823560

C 4.297174 -1.027170 7.884398

C 3.204678 -3.714936 5.389409

C 2.682582 -5.218548 7.252138

C 2.218286 -4.629010 8.577113

C 0.034940 -5.718738 8.600580

C -1.084560 -5.758163 7.563113

C -1.904884 -5.168128 5.387004

C 3.726167 0.134213 8.704095

C 2.992721 1.117313 7.755395

C 2.390855 2.286341 8.547789

C 1.383437 1.739693 9.570341

C 2.109272 0.804613 10.551358

C 2.723464 -0.366960 9.771179

C 3.499170 3.058134 9.273818

C 4.219490 2.107164 10.239809

C 4.820726 0.934184 9.448460

C 3.218729 1.576906 11.275845

C 7.775328 -2.422889 7.045393

C -2.929868 -6.387701 0.620964

C -1.928926 -3.091585 9.384315

C -0.976238 -3.598813 10.297021

C -1.350403 -3.977518 11.588264

C -2.664174 -3.858663 12.025291

C -3.595656 -3.339654 11.118638

C -3.283494 -2.945593 9.815154

C 0.470422 -3.676597 9.904183

C -3.078291 -4.258488 13.417709

C -4.356231 -2.345446 8.903557

C -5.744614 -2.274434 9.580264

C -6.795645 -1.660808 8.640921

C -6.923268 -2.516159 7.372558

C -5.560076 -2.577465 6.672034

C -4.523336 -3.195341 7.620781

C -6.370368 -0.237559 8.255363

C -5.005825 -0.295488 7.556715

C -3.957195 -0.903158 8.499321

C -5.109580 -1.159374 6.290011

C 0.327334 -6.172366 5.615303

C 1.570014 -5.451314 5.098878

C -4.732678 0.594336 2.197108

C -4.072662 1.532885 1.202189

C -2.299105 3.113327 1.116907

C -1.243121 3.768748 1.792043

C -0.492626 4.725148 1.112894

C -0.776318 5.039481 -0.219736

C -1.808735 4.393947 -0.880589

C -2.568796 3.429441 -0.211033

C 0.052272 4.005940 3.778113

C 0.105257 3.460921 5.183454

C -1.117179 3.422890 7.203261

C -2.372233 3.954155 7.850453

C -4.736295 3.865175 7.563361

C -5.822947 3.578514 6.705030

C -7.120151 3.861988 7.121087

C -7.352512 4.463453 8.362083

C -6.285609 4.779614 9.187740

C -4.979875 4.478009 8.789577

C -6.566015 2.644728 4.645181

C -6.008720 2.290183 3.286619

H -0.115590 -6.714743 4.771494

H 0.613452 -6.933653 6.348362

H 2.313547 -6.213561 4.802097

H 1.315155 -4.882577 4.203865

H 3.778983 -5.198458 7.244395

H 2.399298 -6.273812 7.189717

H 2.568576 -5.285697 9.394461

H 2.689280 -3.654438 8.723595

H -0.414527 -5.939723 9.576042

H 0.752005 -6.523003 8.408910

H -1.465602 -6.793768 7.504243

H -1.908948 -5.127929 7.899547

H -2.510174 -4.361544 5.822732

H -2.501999 -6.088622 5.495074

H -1.377095 -4.239725 0.146268

H -2.942141 -6.564086 3.358639

H 3.417889 -1.495162 1.815235

H -0.043487 1.044960 2.088236

H 0.803991 -1.054551 -1.572994

H 1.590280 -2.631990 3.055159

H 1.935154 -3.485943 1.544756

H 0.403753 -3.208989 -0.437937

H -1.016953 -2.165603 -0.326656

H 1.854500 -0.111027 3.198271

H 2.425091 0.794443 1.789245

H -0.726309 0.370243 -0.218579

H 0.893574 1.066350 -0.242602

H -1.526305 -0.937835 1.802066

H -0.491834 -1.093850 3.228718

H 2.926885 -0.432047 -0.401446
H 2.738600 -2.186410 -0.484267
H -2.214290 -6.955487 0.012993
H -3.544243 -5.803006 -0.073939
H -3.587310 -7.110902 1.113192
H 2.695230 -3.083957 4.650029
H 3.862397 -4.394169 4.822126
H 6.331514 -0.608162 8.409549
H 5.872611 -3.805247 5.629885
H 5.031489 2.642605 10.750475
H 1.876429 2.956793 7.847103
H 1.391126 0.411084 11.283168
H 5.552986 1.325788 8.729542
H 5.366494 0.273545 10.136568
H 3.239154 -1.052709 10.458699
H 1.932510 -0.928879 9.270194
H 4.213358 3.470605 8.547621
H 3.077212 3.909384 9.826840
H 0.587351 1.193762 9.047777
H 0.914599 2.566748 10.123571
H 3.711712 1.494012 7.014154
H 2.201897 0.590257 7.211551
H 2.792640 2.410858 11.850723
H 3.730054 0.920845 11.993637
H 8.129344 -2.857886 7.988882
H 8.301554 -1.470351 6.916043
H 8.094991 -3.089689 6.238651
H 0.882944 -2.668653 9.759987
H 1.033997 -4.124164 10.739676
H -4.620035 -3.238972 11.463039
H -0.589219 -4.366449 12.264211
H -7.761578 -1.630234 9.163568
H -5.634836 -3.197253 5.767823
H -4.690190 0.720390 7.286308
H -6.070468 -3.280803 9.878309
H -5.686774 -1.669869 10.495464
H -3.871768 -0.292798 9.409369
H -2.976136 -0.904766 8.011767
H -7.680656 -2.086932 6.701461
H -7.262037 -3.528886 7.631325
H -4.130424 -1.200232 5.793521
H -5.821482 -0.715791 5.580109
H -3.554691 -3.255828 7.120308

H -4.822159 -4.216165 7.898642

H -6.310203 0.396431 9.149947

H -7.124393 0.215028 7.595315

H -3.460432 -3.403056 13.988153

H -2.236292 -4.676016 13.978224

H -3.873320 -5.013849 13.404483

H -0.091039 5.094303 3.806588

H 0.995892 3.781075 3.263701

H 0.192686 2.361555 5.192546

H 1.000740 3.876521 5.673203

H -0.259197 3.811030 7.774193

H -1.073978 2.321212 7.240321

H -2.408746 3.582423 8.882859

H -2.371374 5.052433 7.865341

H -4.154856 4.716885 9.449921

H -6.452954 5.253209 10.150234

H -8.371783 4.679054 8.667248

H -7.964909 3.619764 6.487102

H -7.279599 3.468997 4.507752

H -7.094995 1.788576 5.084041

H -6.852305 2.021412 2.630363

H -5.508505 3.171063 2.858548

H -5.607140 0.136125 1.707471

H -4.042816 -0.207134 2.475221

H -3.716952 0.942620 0.345868

H -4.790165 2.273913 0.821839

H -3.367073 2.924135 -0.742120

H -2.033838 4.625355 -1.917033

H -0.177410 5.788501 -0.728643

H 0.321411 5.233332 1.615587

Complex 4
209

Coordinates

U 4.748521 -5.364434 0.054523

K 3.781391 -1.992502 -7.212601

S 3.954781 -4.390465 -2.251761

O 3.238537 -0.228412 -9.511633

O 1.262369 -2.117262 -8.651019

O 4.570706 -4.706251 -7.589570

O 6.470166 -2.611492 -8.002774

O 4.448878 0.057434 -5.315084

O 2.630128 -2.011009 -4.785452

O 3.161143 -6.983327 -0.011147

O 4.170189 -3.405079 1.063426
O 6.809900 -5.698516 -0.876682
N 1.801136 -4.167388 -6.557139
N 5.776888 0.278569 -8.002281
N 3.965868 -6.223701 2.690560
N 6.176151 -7.523658 1.254472
N 6.464864 -4.754989 2.217775
C 5.352484 0.878605 -9.267505
C 3.850596 1.043904 -9.417162
C 1.863092 -0.137347 -9.828595
C 1.274316 -1.524063 -9.929358
C 0.653879 -3.398079 -8.648813
C 0.527950 -3.900184 -7.221081
C 1.497677 -2.799646 -4.461620
C 1.637504 -4.170028 -5.094188
C 2.365389 -5.442564 -7.005517
C 3.851343 -5.594292 -6.738662
C 5.967474 -4.888549 -7.485318
C 6.664743 -3.945472 -8.436067
C 7.140090 -1.668478 -8.812494
C 7.112386 -0.310047 -8.133432
C 5.749056 1.267991 -6.918667
C 5.700658 0.670046 -5.523030
C 4.366361 -0.567786 -4.034659
C 2.967927 -1.068875 -3.786334
C 5.888496 -4.881090 3.574543
C 4.384907 -5.140173 3.591085
C 5.187964 -8.308790 2.011330
C 4.504061 -7.527762 3.126748
C 2.482368 -6.235495 2.624932
C 2.727674 -10.943147 -3.729156
C 0.913405 -9.345756 -4.392097
C 1.246329 -10.583389 -3.547488
C 0.961316 -10.285076 -2.067027
C 3.298225 -9.484375 -1.771934
C 3.590193 -9.767532 -3.252524
C 3.261520 -8.516106 -4.081447
C 1.774106 -8.169442 -3.914080
C 1.485997 -7.870059 -2.435989
C 1.814364 -9.101989 -1.554142
C -0.777628 -10.060679 2.692401
C 1.870684 -7.415481 1.925157
C 0.913449 -8.178012 2.597895

C 0.260314 -9.235639 1.976610
C 0.596015 -9.500071 0.644075
C 1.543919 -8.775405 -0.082802
C 2.217657 -7.705734 0.584575
C 9.023179 -3.633084 -1.441737
C 10.341628 -2.030594 -2.847671
C 9.063038 -2.211283 -2.018419
C 7.833454 -1.996697 -2.913813
C 7.799711 -4.435254 -3.493890
C 7.852650 -3.011255 -4.067094
C 9.132278 -2.825046 -4.892558
C 10.349773 -3.058673 -3.987347
C 10.294392 -4.479791 -3.404057
C 9.013200 -4.696654 -2.565513
C 11.074560 -9.287829 -1.991893
C 7.813530 -6.514610 -1.171353
C 8.929836 -6.102465 -1.965872
C 9.948046 -7.031413 -2.193214
C 9.942955 -8.338622 -1.694111
C 8.847126 -8.719790 -0.929339
C 7.794700 -7.838806 -0.672227
C 6.602114 -8.341235 0.088524
C 7.317829 -7.119233 2.103364
C 7.640528 -5.625902 2.052422
C 6.866561 -3.355372 1.911211
C 5.880504 0.993419 4.228531
C 4.544521 -2.353235 1.781041
C 5.869307 -2.292165 2.277206
C 6.290051 -1.218302 3.065246
C 5.434058 -0.170662 3.382348
C 4.129956 -0.236100 2.878245
C 3.647005 -1.282180 2.088702
C 0.039730 -2.535587 -0.022636
C 1.422779 -2.494030 2.064831
C -0.010244 -2.511022 1.512533
C -0.756316 -1.256620 1.984689
C 0.765973 -1.279911 -0.528443
C 2.205023 -1.268184 0.012629
C 2.208080 -1.258833 1.562627
C 1.424934 -0.007075 2.021797
C -0.013588 -0.012309 1.478379
C 0.018086 -0.026437 -0.055794
H 8.397545 -5.410480 2.828940

H 8.090029 -5.386239 1.088017

H 6.073896 -3.966412 4.150678

H 6.411754 -5.683592 4.104392

H 7.081348 -3.321983 0.833898

H 7.817984 -3.127983 2.420342

H 3.461498 0.583143 3.123548

H 7.316581 -1.205809 3.431251

H -0.525651 -3.409164 1.878748

H 0.796521 -1.296984 -1.625391

H -0.527366 0.892910 1.830289

H 1.955066 -3.390686 1.741740

H 1.406787 -2.490833 3.164007

H 1.395324 0.036582 3.119406

H 1.934602 0.902969 1.676572

H 0.567898 -3.432522 -0.370378

H -0.979595 -2.576051 -0.431995

H 0.519194 0.877339 -0.429948

H -1.005957 -0.015434 -0.455107

H 2.732679 -0.371047 -0.342541

H 2.755831 -2.140794 -0.356934

H -0.816162 -1.241207 3.081810

H -1.788716 -1.260040 1.607937

H 6.912457 0.863933 4.569186

H 5.251778 1.113607 5.119179

H 5.833866 1.939884 3.675623

H -0.002450 -3.129086 -6.654257

H -0.127913 -4.792752 -7.227385

H 1.235910 -4.090781 -9.277623

H -0.355368 -3.332932 -9.087373

H 0.250243 -1.442862 -10.330068

H 1.863616 -2.127936 -10.639154

H 1.725576 0.380391 -10.792007

H 1.323905 0.434929 -9.057037

H 3.416564 1.612457 -8.579355

H 3.658280 1.623899 -10.334804

H 5.685658 0.236389 -10.088584

H 5.828265 1.865468 -9.426997

H 2.219546 -5.536099 -8.087334

H 1.841591 -6.297049 -6.539705

H 4.102623 -5.402480 -5.685691

H 4.139998 -6.633018 -6.963993

H 6.243237 -5.922169 -7.752301

H 6.311154 -4.706551 -6.456254

H 7.739211 -4.187028 -8.454067
H 6.268164 -4.081676 -9.455437
H 6.686486 -1.636527 -9.817160
H 8.194424 -1.960529 -8.945033
H 7.532172 -0.448604 -7.133675
H 7.800672 0.367123 -8.674425
H 5.656951 -9.208157 2.452082
H 4.434647 -8.655252 1.301133
H 8.222970 -7.659772 1.801040
H 7.116095 -7.421812 3.135979
H 5.729130 -8.409644 -0.574608
H 6.814354 -9.370972 0.422329
H 10.802075 -6.733541 -2.793468
H 8.796835 -9.730379 -0.524413
H 9.049945 -1.485719 -1.193820
H 6.971540 -2.855352 -4.704449
H 11.273450 -2.951770 -4.573045
H 8.123716 -3.765747 -0.837365
H 9.892407 -3.804149 -0.791100
H 11.185622 -4.644647 -2.782879
H 10.334937 -5.209847 -4.224240
H 6.911586 -2.123931 -2.332761
H 7.836077 -0.969193 -3.307573
H 9.148467 -3.531584 -5.734652
H 9.175094 -1.812197 -5.321217
H 7.823236 -5.170518 -4.311734
H 6.859053 -4.582551 -2.952256
H 11.227600 -2.165604 -2.212145
H 10.393479 -1.011439 -3.256469
H 10.975707 -10.215674 -1.419986
H 12.048828 -8.849425 -1.745133
H 11.107636 -9.561450 -3.054242
H 4.082611 -5.354518 4.633135
H 3.858161 -4.236453 3.281966
H 3.700856 -8.158163 3.528030
H 5.202528 -7.366891 3.954354
H 2.184525 -5.304709 2.125794
H 2.069302 -6.185777 3.646868
H 0.077483 -10.319814 0.156456
H 0.673140 -7.926520 3.630725
H 4.653909 -10.016399 -3.367686
H 1.538542 -7.272926 -4.501497
H 0.619834 -11.428266 -3.865346

H 3.919569 -8.656546 -1.424333

H 3.539532 -10.366372 -1.161495

H 1.168173 -11.184594 -1.470462

H -0.106604 -10.057732 -1.946246

H 3.875106 -7.668667 -3.749966

H 3.492286 -8.702532 -5.141057

H -0.153193 -9.099607 -4.295209

H 1.099928 -9.554500 -5.455572

H 0.425253 -7.614558 -2.303344

H 2.076331 -7.006940 -2.110643

H 2.969924 -11.848371 -3.155184

H 2.935327 -11.168476 -4.784749

H -0.869303 -9.761160 3.741003

H -0.531034 -11.129173 2.674358

H -1.768489 -9.954343 2.233464

H 2.515271 -4.618962 -4.616618

H 0.765065 -4.781303 -4.796228

H 0.577666 -2.273443 -4.768976

H 1.457277 -2.971259 -3.378390

H 2.970963 -1.554902 -2.801643

H 2.251914 -0.230121 -3.785532

H 4.629467 0.152430 -3.244726

H 5.065449 -1.413383 -3.973804

H 6.514970 -0.055036 -5.366067

H 5.856387 1.482183 -4.793067

H 4.853019 1.885752 -7.033308

H 6.615864 1.952978 -6.982315

Complex 4 without counter-ion
146

Coordinates

U -0.334123 2.528374 6.589295

S 2.126590 1.945795 6.085461

O -0.734435 0.384993 7.218628

O 0.338056 4.032283 8.145825

O -0.642228 3.169897 4.434211

N -3.091520 1.694029 6.162025

N -2.280622 2.930901 8.735869

N -2.389651 4.574876 6.274407

C 2.842553 -0.634603 9.431853

C 0.368729 -0.935018 9.723901

C 1.727129 -0.950142 10.441275

C 1.964244 -2.336560 11.055385

C 1.497567 -1.677045 7.594968

C 2.848227 -1.699773 8.325423

C 3.085111 -3.084676 8.941399

C 1.956404 -3.389111 9.937446

C 0.602631 -3.359654 9.209416

C 0.332724 -1.975413 8.576800

C -3.871896 -4.328407 6.975038

C -1.415951 -0.743777 7.137184

C -1.000836 -1.925822 7.825623

C -1.826689 -3.048372 7.738215

C -3.016585 -3.087706 6.999830

C -3.369994 -1.942017 6.295024

C -2.587906 -0.786023 6.344048

C -2.948428 0.386228 5.473440

C -0.098592 6.413882 13.269409

C 1.781293 6.605296 7.463047

C 3.766057 8.106484 7.166457

C 2.933908 6.988734 6.523253

C 3.822223 5.759302 6.273529

C 3.257345 4.870534 8.545024

C 4.405445 5.270384 7.608004

C 5.236195 6.389408 8.250092

C 4.330544 7.604067 8.502951

C 3.175680 7.202891 9.435817

C 2.317391 6.075379 8.816244

C 0.295447 4.570423 9.351314

C 1.177192 5.628436 9.735214

C 1.004486 6.185672 11.003412

C 0.042496 5.749218 11.924100

C -0.763500 4.679787 11.548573

C -0.644394 4.083555 10.291049

C -1.459343 2.860376 9.970540

C -3.667872 4.103587 5.708396

C -3.635275 2.663446 5.201990

C -3.921664 1.589952 7.377043

C -3.143109 1.743267 8.682002

C -3.056784 4.182812 8.667514

C -2.561573 5.163815 7.608592

C -1.737043 5.584138 5.402094

C -2.544214 6.898563 0.612165

C -1.316983 4.774962 1.237228

C -1.995854 5.934596 1.632772

C -2.139908 6.156018 2.998148

C -1.657525 5.243792 3.939166

C -1.022713 4.048393 3.523572

C -0.808225 3.827371 2.127838

C 0.164669 2.615347 0.096538

C 1.436834 2.689991 2.244770

C 0.010361 2.631140 1.634780

C -0.650578 1.289692 2.039253

C 1.011496 1.420415 -0.370984

C 2.411588 1.505065 0.253210

C 2.278962 1.492216 1.781699

C 1.597119 0.191002 2.230503

C 0.203863 0.094674 1.589479

C 0.337243 0.109849 0.060561

H -3.871725 1.745378 9.516135

H -2.500610 0.870932 8.817529

H -3.023609 4.702789 9.633452

H -4.111123 3.943086 8.492125

H -0.799860 1.988576 9.856606

H -2.112793 2.644032 10.834721

H 1.656896 6.998578 11.309570

H -1.499440 4.285346 12.249837

H 2.515069 7.341086 5.570403

H 5.031126 4.388086 7.426347

H 4.909865 8.405838 8.983789

H 1.172190 5.826440 6.999734

H 1.133820 7.476026 7.646299

H 2.551056 8.084860 9.640723

H 3.591299 6.871373 10.397559

H 3.243769 4.947235 5.815408

H 4.633671 6.019926 5.578375

H 5.673455 6.043312 9.197586

H 6.072737 6.674892 7.595753

H 3.660170 4.517151 9.505125

H 2.698923 4.042865 8.095980

H 3.144097 8.998721 7.330914

H 4.586740 8.406384 6.499101

H -0.772776 5.849585 13.922117

H -0.502292 7.431794 13.184984

H 0.866091 6.496956 13.784524

H -4.663239 2.384637 4.898336

H -3.011711 2.615720 4.306999

H -4.421217 0.613362 7.411859

H -4.722704 2.335672 7.327905

H -2.183233 0.526653 4.696591

H -3.886778 0.146122 4.942110

H -1.534462 -3.954465 8.261050

H -4.269846 -1.942329 5.679315

H 1.721077 -0.190226 11.235020

H 3.638672 -1.464213 7.602542

H 2.102723 -4.389013 10.371501

H 0.183879 0.055403 9.302252

H -0.440411 -1.153562 10.436742

H -0.196391 -3.612971 9.921687

H 0.596916 -4.133780 8.429452

H 2.689966 0.352745 8.978937

H 3.815426 -0.614521 9.944365

H 3.108571 -3.851781 8.154275

H 4.057620 -3.119765 9.453713

H 1.487959 -2.434199 6.797974

H 1.360770 -0.699022 7.122039

H 1.181708 -2.564585 11.793839

H 2.925594 -2.359862 11.588257

H -4.624687 -4.276559 6.181496

H -4.407481 -4.478268 7.922356

H -3.272342 -5.230194 6.803181

H -3.266591 6.017464 7.575538

H -1.591859 5.561376 7.914918

H -3.962604 4.735796 4.861105

H -4.457597 4.220331 6.458286

H -0.727386 5.739707 5.807845

H -2.262557 6.550472 5.502933

H -1.174654 4.620317 0.171720

H -2.629010 7.064808 3.349879

H -0.286403 -0.835557 1.908687

H 3.268397 1.560325 2.249868

H 1.090282 1.450788 -1.467581

H -0.769140 1.271625 3.124643

H -1.653961 1.230655 1.591488

H -0.824000 2.567052 -0.383427

H 0.640756 3.547005 -0.239766

H 1.524282 0.183148 3.325041

H 2.204461 -0.676856 1.934680

H 2.914919 2.424812 -0.077279

H 3.027465 0.660653 -0.089009

H 1.914220 3.629264 1.930852

H 1.387857 2.695102 3.339022

H -0.653160 0.022408 -0.409668

H 0.931822 -0.749794 -0.280313

H -2.807373 7.857066 1.071741

H -3.450061 6.512079 0.125350

H -1.818023 7.101871 -0.183610

Complex 2
147

Coordinates

U 4.734381 5.379172 0.097368

S 2.376335 6.466788 1.223044

O 5.600320 7.017848 1.331588

O 4.173305 3.452794 1.058756

O 3.751941 5.757981 -1.863619

N 6.997158 4.076093 0.845151

N 6.997308 6.279998 -1.082323

N 5.785851 3.708357 -1.791601

C -0.026260 0.143996 -0.024934

C 0.005036 0.032665 1.505465

C 1.463509 0.011647 1.993134

C 2.212401 1.302296 1.587273

C 2.141409 1.403639 0.044005

C 0.685591 1.435793 -0.446278

C -0.728297 1.231873 2.122735

C -0.014243 2.524297 1.705985

C 1.439241 2.496240 2.205432

C -0.033333 2.643638 0.174295

C 3.665674 1.316795 2.074597

C 4.538391 2.414109 1.820358

C 5.835949 2.414651 2.376464

C 6.286462 1.325815 3.127609

C 5.470850 0.225489 3.362456

C 4.175804 0.258914 2.832133

C 6.721273 3.622242 2.241662

C 8.046091 5.115493 0.889997

C 8.257128 5.816035 -0.448528

C 6.886177 7.771301 -1.041348

C 7.187196 8.412381 0.283836

C 6.459026 8.040177 1.433763

C 6.666238 8.743325 2.655719

C 7.638210 9.748042 2.652287

C 8.393627 10.109400 1.530358

C 8.144176 9.428779 0.344941

C 5.835135 8.463346 3.913388

C 6.254728 9.351870 5.108001

C 5.393382 9.067920 6.348922

C 3.918994 9.353837 6.029286

C 3.477006 8.457648 4.865260

C 4.338746 8.753507 3.628066

C 5.553768 7.598822 6.763322

C 5.110407 6.700570 5.601447

C 5.979786 6.989773 4.368209

C 3.638143 6.982438 5.263169

C 9.421556 11.207546 1.613768

C 5.944113 -0.955853 4.168654

C 3.600774 5.450098 -3.157322

C 4.073356 4.195403 -3.595084

C 3.978775 3.825677 -4.938671

C 3.410062 4.678580 -5.877271

C 2.928584 5.911498 -5.420795

C 2.990846 6.337384 -4.090385

C 4.621488 3.221005 -2.593883

C 6.330641 2.556867 -1.043341

C 7.396656 2.943686 -0.025658

C 2.386290 7.682183 -3.669486

C 1.785496 8.458338 -4.864080

C 1.174100 9.794992 -4.413727

C 0.055243 9.530316 -3.396536

C 0.641229 8.788710 -2.187769

C 1.247093 7.451814 -2.643083

C 2.258924 10.669199 -3.768991

C 2.844468 9.928356 -2.560390

C 3.456625 8.597690 -3.024540

C 1.733835 9.649432 -1.535768

C 3.303701 4.301236 -7.331899

C 6.907935 5.847431 -2.492870

C 6.815184 4.334154 -2.657142

H 8.922742 6.668863 -0.275229

H 8.780146 5.154735 -1.145260

H 7.777622 6.212250 -3.065284

H 6.019184 6.315196 -2.924665

H 7.562902 8.196392 -1.798540

H 5.866018 8.019463 -1.366046

H 7.823968 10.293329 3.571526

H 8.691727 9.695565 -0.558292

H 5.729362 9.720559 7.166059

H 2.427045 8.654456 4.615912

H 5.229766 5.645329 5.882361

H 7.312448 9.180422 5.353384

H 6.152675 10.412084 4.839792

H 5.675146 6.338165 3.545543

H 7.036487 6.780443 4.590031

H 3.297893 9.166186 6.915896

H 3.788138 10.412199 5.765327

H 3.302624 6.339768 4.439948

H 3.004428 6.754812 6.131333

H 3.993268 8.150492 2.784715

H 4.231187 9.807757 3.338350

H 4.951667 7.390223 7.657992

H 6.600752 7.392195 7.026225

H 8.963489 12.168263 1.876969

H 10.179297 10.994741 2.376986

H 9.940753 11.341065 0.660080

H 6.595483 4.127566 -3.710633

H 7.783633 3.869671 -2.449505

H 6.757284 1.810839 -1.735415

H 5.494183 2.080732 -0.527736

H 4.914740 2.299729 -3.120690

H 3.846131 2.935859 -1.870757

H 2.475095 6.568193 -6.155832

H 4.348640 2.848495 -5.246946

H 0.761627 10.306810 -5.293656

H -0.149207 8.586612 -1.453802

H 3.629971 10.539532 -2.095778

H 2.561983 8.651373 -5.617630

H 1.006322 7.854590 -5.348583

H 3.893643 8.077321 -2.168771

H 4.260433 8.781599 -3.752327

H -0.398549 10.479311 -3.079909

H -0.741992 8.932780 -3.859704

H 2.140339 9.129782 -0.659516

H 1.305339 10.597493 -1.182809

H 1.639274 6.911360 -1.776917

H 0.473527 6.822964 -3.105197

H 1.832007 11.631608 -3.456231

H 3.049234 10.891570 -4.499717

H 3.885791 4.978007 -7.969007

H 3.670000 3.285596 -7.508466

H 2.266519 4.344476 -7.683503

H 1.441965 5.699587 0.603068

H 7.612822 2.059243 0.584446

H 8.331048 3.201116 -0.533223

H 9.007079 4.683214 1.216856

H 7.748119 5.848834 1.641816

H 7.678848 3.411861 2.742495

H 6.281546 4.481620 2.766406

H 3.535099 -0.593408 3.032846

H 7.292363 1.351637 3.544927

H -0.483151 -0.900283 1.817468

H -0.523197 3.390412 2.147839

H 0.677642 1.524072 -1.540985

H 1.971553 -0.868091 1.573717

H 1.474368 -0.098851 3.085729

H 2.656610 2.314175 -0.272039

H 2.672790 0.549393 -0.399562

H -1.774742 1.246702 1.788602

H -0.742959 1.143791 3.217415

H 0.458227 3.575043 -0.136973

H -1.068871 2.693211 -0.188481

H 1.942479 3.435763 1.959205

H 1.457257 2.400974 3.299514

H -1.064584 0.146144 -0.382991

H 0.467785 -0.726264 -0.478537

H 5.325894 -1.107159 5.061253

H 5.900885 -1.884954 3.588065

H 6.977314 -0.822412 4.502549

Model of complex 2 with tacn ligand

129

Coordinates

U 0.764512 2.353379 6.320485

S 3.399265 1.794989 5.730049

O -0.197961 0.510582 6.819318

O 0.937381 3.732083 7.947463

O -0.093730 3.124799 4.515169

C -2.734967 0.987635 5.768392

C -4.151584 -3.490248 7.589037

C 0.670642 -3.260450 9.109376

C 2.406591 -2.393069 10.700030

C 2.066234 -3.508634 9.702211

C 3.108839 -3.529802 8.576039

C 1.679921 -1.953919 7.247531

C 3.078645 -2.182867 7.842707

C 3.411663 -1.055726 8.833158

C 2.377831 -1.044669 9.968899

C 0.975921 -0.808425 9.386542

C 0.601381 -1.907931 8.359665

C -2.406035 -0.273139 6.515617

C -3.381603 -1.248817 6.711010

C -3.105526 -2.421120 7.409622

C -1.816593 -2.586016 7.920139

C -0.788865 -1.646897 7.761987

C -1.112250 -0.473951 7.036597

C -1.313987 2.897534 9.360534

C 3.413605 7.184628 9.407751

C 4.195288 8.111635 7.213239

C 4.637707 7.665435 8.613396

C 5.656510 6.523753 8.496242

C 3.778740 4.861984 8.593031

C 4.996272 5.337864 7.782131

C 4.545822 5.774669 6.379440

C 3.535689 6.926388 6.496445

C 2.311220 6.458247 7.298942

C 2.718700 5.986428 8.718041

C -0.319850 6.494826 12.743732

C -0.456669 4.037165 9.831199

C -0.775198 4.720081 11.003193

C 0.004549 5.780395 11.457773

C 1.114430 6.146748 10.693977

C 1.489304 5.507480 9.504644

C 0.672760 4.429446 9.086315

C -1.575302 5.170817 5.674191

C -3.036231 6.516025 1.043555

C -0.789018 3.901832 3.642493

C -1.549857 4.954735 4.188584

C -2.281588 5.783752 3.341420

C -2.274595 5.595886 1.961420

C -1.518516 4.538574 1.452236

C -0.762743 3.665900 2.246541

C -0.139210 2.485541 0.064832

C 1.553398 2.685355 1.876757

C 0.036636 2.523556 1.602727

C -0.434257 1.149224 2.143258

C 0.661421 1.337865 -0.569510

C 2.153943 1.517947 -0.261752

C 2.352560 1.525799 1.260034

C 1.862451 0.189594 1.839784

C 0.372135 -0.000307 1.519862

C 0.176369 -0.002480 -0.002069

H -2.153771 2.732745 10.040202

H -0.758751 1.953715 9.298973

H 1.710386 6.978208 11.052670

H -1.652261 4.410941 11.568922

H 5.090337 8.512852 9.145220

H 5.709425 4.508523 7.695518

H 3.208373 7.234448 5.494608

H 2.705599 8.017463 9.513689

H 3.730539 6.898351 10.419393

H 1.812798 5.640366 6.768114

H 1.579765 7.272866 7.386926

H 6.540814 6.860607 7.938709

H 6.002954 6.221786 9.493882

H 4.103190 4.924241 5.845344

H 5.415287 6.098240 5.791447

H 3.339370 3.981374 8.115938

H 4.092015 4.553508 9.599386

H 5.059376 8.470061 6.638158

H 3.489748 8.950276 7.289118

H 0.175681 6.020925 13.600492

H 0.008622 7.538290 12.716162

H -1.395221 6.486468 12.946886

H -3.767338 0.970848 5.409996

H -2.088323 1.138218 4.896862

H -1.613626 -3.498189 8.469751

H -4.378244 -1.082488 6.306123

H 2.060363 -4.477407 10.219072

H 3.815609 -2.184222 7.029303

H 2.611708 -0.235832 10.673616

H -0.072295 -3.278099 9.918012

H 0.421216 -4.079947 8.422307

H 0.938824 0.174201 8.905078

H 0.226519 -0.796685 10.189434

H 4.108797 -3.717060 8.989797

H 2.892845 -4.348607 7.876458

H 3.423750 -0.090848 8.310580

H 4.418940 -1.204837 9.244679

H 1.668725 -1.020823 6.676786

H 1.431098 -2.760782 6.545013

H 3.398061 -2.566294 11.139251

H 1.684455 -2.391842 11.527835

H -4.015490 -4.030674 8.530951

H -5.160911 -3.067336 7.587049

H -4.108663 -4.231529 6.780994

H 3.249017 0.957877 4.670486

H -2.237162 5.998782 5.940247

H -0.584133 5.409968 6.079867

H -1.527112 4.395073 0.377789

H -2.867939 6.592199 3.774389

H 0.503438 1.357747 -1.655812

H 3.416300 1.661875 1.494705

H 0.022352 -0.952024 1.940681

H -1.201152 2.366356 -0.187892

H 0.192465 3.437157 -0.371211

H -0.328137 1.125565 3.232447

H -1.503272 1.024474 1.925417

H 2.736035 0.706682 -0.719218

H 2.519986 2.458424 -0.694627

H 2.015646 0.167745 2.928724

H 2.448279 -0.640848 1.423889

H 1.738629 2.734139 2.953672

H 1.892702 3.641342 1.456651

H 0.733753 -0.832177 -0.457077

H -0.883290 -0.156415 -0.246267

H -2.432972 7.388006 0.760733

H -3.326553 6.009162 0.118249

H -3.946598 6.893775 1.519452

H -2.635303 1.876786 6.406774

H -1.745336 3.093024 8.368238

H -1.946378 4.283735 6.206940

References

1. G. A. Bain and J. F. Berry, *J. Chem. Educ.*, 2008, **85**, 532.
2. K. Gademann, D. E. Chavez and E. N. Jacobsen, *Angew. Chem. Int. Ed.*, 2002, **41**, 3059-3061.
3. M. J. Monreal, R. K. Thomson, T. Cantat, N. E. Travia, B. L. Scott and J. L. Kiplinger, *Organometallics*, 2011, **30**, 2031-2038.
4. *SADABS*, 2008, Bruker AXS, Inc., Madison WI., U.S.A.
5. *SHELXTL NT 6.12*, 2002, Bruker AXS, Inc., Madison, WI, U.S.A.
6. G. Sheldrick, *Acta Crystallogr. Sect. A*, 2008, **64**, 112-122.
7. O. V. Dolomanov, L. J. Bourhis, R. J. Gildea, J. A. K. Howard and H. Puschmann, *J. Appl. Crystallogr.*, 2009, **42**, 339-341.
8. S. Parsons, H. D. Flack and T. Wagner, *Acta Crystallogr. Sect. B*, 2013, **69**, 249-259.
9. D. R. English, D. N. Hendrickson, K. S. Suslick, C. W. Eigenbrot and W. R. Scheidt, *J. Am. Chem. Soc.*, 1984, **106**, 7258-7259.
10. T. R. Gaffney and J. A. Ibers, *Inorg. Chem.*, 1982, **21**, 2857-2859.
11. M. M. Ibrahim, J. Seebacher, G. Steinfeld and H. Vahrenkamp, *Inorg. Chem.*, 2005, **44**, 8531-8538.
12. J. W. Pavlik, B. C. Noll, A. G. Oliver, C. E. Schulz and W. R. Scheidt, *Inorg. Chem.*, 2010, **49**, 1017-1026.
13. M. Rombach and H. Vahrenkamp, *Inorg. Chem.*, 2001, **40**, 6144-6150.
14. J. Ruiz, V. Rodríguez, C. Vicente, J. M. Martí, G. López and J. Pérez, *Inorg. Chem.*, 2001, **40**, 5354-5360.
15. F. Secheresse, J. M. Manoli and C. Potvin, *Inorg. Chem.*, 1986, **25**, 3967-3971.
16. S. M. Franke, M. W. Rosenzweig, F. W. Heinemann and K. Meyer, *Chem. Sci.*, 2015, **6**, 275-282.
17. Gaussian09, Revision A.02, M. J. Frisch, G. W. Trucks, H. B. Schlegel, G. E. Scuseria, M. A. Robb, J. R. Cheeseman, G. Scalmani, V. Barone, B. Mennucci, G. A. Petersson, H. Nakatsuji, M. Caricato, X. Li, H. P. Hratchian, A. F. Izmaylov, J. Bloino, G. Zheng, J. L. Sonnenberg, M. Hada, M. Ehara, K. Toyota, R. Fukuda, J. Hasegawa, M. Ishida, T. Nakajima, Y. Honda, O. Kitao, H. Nakai, T. Vreven, J. A. Montgomery, Jr., J. E. Peralta, F. Ogliaro, M. Bearpark, J. J. Heyd, E. Brothers, K. N. Kudin, V. N. Staroverov, R. Kobayashi, J. Normand, K. Raghavachari, A. Rendell, J. C. Burant, S. S. Iyengar, J. Tomasi, M. Cossi, N. Rega, J. M. Millam, M. Klene, J. E. Knox, J. B. Cross, V. Bakken, C. Adamo, J. Jaramillo, R. Gomperts, R. E. Stratmann, O. Yazyev, A. J. Austin, R. Cammi, C. Pomelli, J. W. Ochterski, R. L. Martin, K. Morokuma, V. G. Zakrzewski, G. A. Voth, P. Salvador, J. J. Dannenberg, S. Dapprich, A. D. Daniels, O. Farkas, J. B. Foresman, J. V. Ortiz, J. Cioslowski, D. J. Fox, Gaussian, Inc., Wallingford CT, 2009.
18. A. D. Becke, *J. Chem. Phys.*, 1993, **98**, 5648-5652.
19. J. P. Perdew and Y. Wang, *Phys. Rev. B*, 1992, **45**, 13244-13249.
20. X. Cao and M. Dolg, *J. Mol. Struct.: THEOCHEM*, 2004, **673**, 203-209.
21. X. Cao, M. Dolg and H. Stoll, *J. Chem. Phys.*, 2003, **118**, 487-496.
22. W. Kuechle, M. Dolg, H. Stoll and H. Preuss, *J. Chem. Phys.*, 1994, **100**, 7535-7542.
23. T. Leininger, A. Nicklass, W. Kuechle, H. Stoll, M. Dolg and A. Bergner, *Chem. Phys. Lett.*, 1996, **255**, 274-280.
24. A. Bergner, M. Dolg, W. Kuechle, H. Stoll and H. Preuss, *Mol. Phys.*, 1993, **80**, 1431-1441.
25. A. W. Ehlers, M. Boehme, S. Dapprich, A. Gobbi, A. Hoellwarth, V. Jonas, K. F. Koehler, R. Stegmann, A. Veldkamp and a. et, *Chem. Phys. Lett.*, 1993, **208**, 111-114.
26. W. J. Hehre, R. Ditchfield and J. A. Pople, *J. Chem. Phys.*, 1972, **56**, 2257-2261.
27. P. C. Hariharan and J. A. Pople, *Theor. Chim. Acta*, 1973, **28**, 213-222.
28. H. B. Schlegel, *J. Comput. Chem.*, 1982, **3**, 214-218.

29. G. A. Zhurko, *Home Page: a set of graphical tools for facilitating working with quantum chemistry computations (*http://www.chemcraftprog.com*)*.
